# Multidisciplinary stakeholder-informed identification of key characteristics for implementation of workplace genetic testing

**DOI:** 10.1016/j.xhgg.2025.100458

**Published:** 2025-05-22

**Authors:** Elizabeth Charnysh, Kunal Sanghavi, Kerry A. Ryan, Alyx Vogle, Alexandra Truhlar, Subhamoy Pal, Jonathan M. Reader, J. Scott Roberts, Charles Lee, Anya E.R. Prince, W. Gregory Feero, INSIGHT @ Work Consortium

**Affiliations:** 1 The Jackson Laboratory for Genomic Medicine, Farmington, CT, USA; 2 Center for Bioethics and Social Sciences in Medicine, University of Michigan School of Medicine, Ann Arbor, MI, USA; 3 Department of Cardiology, Brigham and Women’s Hospital, Boston, MA, USA; 4 Michigan Alzheimer’s Disease Research Center, Department of Neurology, University of Michigan School of Medicine, Ann Arbor, MI, USA; 5 Department of Health Behavior and Health Equity, University of Michigan School of Public Health, Ann Arbor, MI, USA; 6 University of Iowa College of Law, Iowa City, IA, USA; 7 Maine Dartmouth Family Medicine Residency, Augusta, ME, USA

**Keywords:** ELSI, genetic testing, workplace genetic testing, wellness programs, Delphi

## Abstract

Workplace genetic testing (wGT) is an evolving model for genetic testing where employees are offered consumer genetic testing through employer-sponsored wellness programs. However, the potential harms, benefits, and key characteristics for best implementation practices for wGT have yet to be defined. To address this issue, we conducted a three-round modified Delphi process, including multiple rounds of survey and a virtual deliberative workshop, with purposely chosen wGT stakeholders (employees, employers, ethical, legal, and social implications [ELSI] professionals, genetic testing industry representatives, and healthcare professionals) to share their perspectives. From the modified Delphi process, we identified 12 key characteristics for the implementation of wGT that were perceived to increase the potential for benefit while reducing the risk of potential harms. Most participants agreed that privacy/security, voluntariness, transparency, understanding and education, anti-discrimination, employee control, and evidence-based testing measures were both important (>90%) and necessary (>75%) for the implementation of wGT. However, some participants also expressed a lack of confidence in the likelihood of achieving these characteristics in wGT programs. Overall, stakeholders expressed qualified support for wGT at the conclusion of the modified Delphi process. Their perspectives on the topic varied over the course of the process and were at least partially contingent on whether the aforementioned 12 key characteristics were met. These findings help inform the establishment of a normative framework for wGT assessment.

## Teaser

Workplace genetic testing is a model where employees are offered genetic testing through wellness programs. However, the potential harms, benefits, and best implementation practices have yet to be defined. From a modified Delphi process, we identified 12 key characteristics for implementation. These findings help inform the establishment of a normative framework.

## Introduction

Clinical, public, and private predictive genetic testing service providers have long had the goal of increasing access to genetic testing given its potential to inform disease prevention and screening.^1^^,^^2^^,^^3^ Avenues for genetic testing have shifted dramatically over the past few decades, from testing primarily taking place in clinical settings,^4^ to now having consumer genetic testing options outside healthcare systems, such as direct-to-consumer genetic testing (dtcGT), where consumers order testing directly without involvement of a clinician,^4^ and consumer-initiated genetic testing (ciGT), where the laboratory assigns a clinician to be the ordering provider for a consumer’s test order.^5^ More recently, the workplace has emerged as another access point for consumer genetic testing, where genetic testing is offered through employer-sponsored wellness programs, otherwise known as workplace genetic testing (wGT).^6^^,^^7^^,^^8^^,^^9^^,^^10^ Some major companies have shared publicly that they have implemented wGT.^6^^,^^11^^,^^12^^,^^13^^,^^14^ While one study identified lack of evidence of broad uptake of wGT in the United States,^11^ it has been challenging for researchers to fully analyze the evolving landscape.^11^ Typically, wGT is offered at no cost to employees, is performed by a consumer genetic testing laboratory, and includes multi-gene panel testing for a range of conditions and purposes (e.g., hereditary cancer syndromes, hereditary cardiovascular disease, and medication response [pharmacogenomics]).^9^ One study examining employees of a large US healthcare system reported that approximately half of survey respondents indicated they pursued wGT and received their test results, and that those who received results indicating they were at increased risk for cancer or heart disease were more likely to change their health behaviors or utilize healthcare services.^9^ However, other studies published to date have mostly explored employees’ perspectives on a hypothetical wGT scenario.^7^^,^^15^^,^^16^

As genetic testing moves into the workplace, it is important to examine the potential benefits and harms in this context. While there have been limited studies on wGT specifically, previous research on consumer genetic testing can offer insights into the potential benefits and harms of wGT. Consumer genetic testing has been shown to increase awareness and access to genetic testing for those who may not otherwise meet clinical criteria or have ready access to clinical genetics services,^17^^,^^18^^,^^19^ as well as increase the overall ease of use of genetic testing.^3^ However, consumer genetic testing is only available to those who have the means to pay for it, raising concerns about the potential for worsening socioeconomic disparities.^20^^,^^21^^,^^22^^,^^23^ A myriad of other challenges with consumer genetic testing have been identified, including the lack of genetic counseling typically included in consumer genetic testing workflows,^24^^,^^25^ false reassurance and confusion about risk information,^3^^,^^26^^,^^27^ the mixing of medical findings with ancestry and non-medical information,^28^ integration of results into the medical record and appropriate follow-up care,^29^ and privacy and secondary data sharing/use concerns.^30^^,^^31^^,^^32^

In general, workplace wellness programs have their own set of ethical, legal, and social implications (ELSI). Often touted as a “win-win”^33^ for the employer and employee given their potential to expand access to actionable health information, these programs have raised questions and concerns about effectiveness,^34^^,^^35^^,^^36^^,^^37^ privacy and data sharing,^38^^,^^39^^,^^40^ and whether incentives are truly rewards (“carrots”), or actually discriminatory penalties (“sticks”).^33^^,^^40^^,^^41^ Introducing genetic testing as a wellness benefit adds additional complexity to these programs given the sensitivity and nature of genetic information as well as the nuanced policies and laws surrounding it. For example, while the Genetic Information Nondiscrimination Act (GINA) generally prohibits employers from collecting genetic information from their employees and discriminating against them based on genetic information, GINA has an exception allowing employers to collect employee genetic information in a deidentified, aggregate manner as part of workplace wellness programs.^6^

Currently, there are no policy guidelines for the assessment and implementation of wGT.^6^ One recent study reported on programmatic design features that could reduce barriers to employee participation in a hypothetical wGT program based on a survey of employed US adults.^16^ Briscoe and colleagues identified the ability to later delete one’s data from the wGT program, policies prohibiting data sale or sharing, and control over how employee data are used to be design features most likely to increase respondents’ likelihood of participating in a theoretical wGT program.^16^ Existing frameworks for ethical implementation of consumer genetic testing have prioritized informed consent, respect for privacy and confidentiality, genetic counseling, and ensuring clinical utility and validity, among other criteria.^42^^,^^43^^,^^44^ However, understanding which key characteristics are important to the ethical design and implementation of a wGT program from the perspectives of stakeholders is critical. Key characteristics, as identified by stakeholders directly involved with or affected by wGT, can inform policy deliberations, normative assessment, and wGT program implementation. To this end, we conducted a modified Delphi process with multiple survey rounds and a deliberative workshop to assess acceptance of wGT from the perspectives of important stakeholders and to identify key characteristics that have the potential to enhance benefit of wGT to individuals and society.

## Methods

### Participants, recruitment, and study design

Important stakeholder groups were identified by the research team (E.C., W.G.F., A.E.R.P., K.A.R., K.S., and A.V.) and through review of relevant literature.^6^ Stakeholder groups included (1) employees (full-time workers and/or labor organization representatives), (2) employers (managers of companies and/or business representatives), (3) ELSI professionals (bioethics, legal, privacy, or policy experts), (4) genetic testing industry representatives (commercial genetic testing laboratory representatives, commercial genetic counseling service providers, and health insurance industry professionals), and (5) healthcare professionals (genetic counselors, physicians, nurses, other clinicians). Consistent with similar studies involving Delphi methodology,^45^^,^^46^^,^^47^^,^^48^^,^^49^^,^^50^^,^^51^ we aimed to recruit 40–50 research participants for the study. Potential individuals representing each stakeholder group were initially identified by the research team with the input of an expert scientific advisory board composed of ELSI experts, followed by snowball sampling. Participants were then prioritized for enrollment based on personal characteristics such as years of experience, education, race and ethnicity, and gender collected through the study screener to try to maximize the diversity of perspectives represented within stakeholder groups. Participants provided written consent before participation. Participants received a total of $300 for their full participation.

A modified Delphi process was carried out from June 2023 to April 2024 (Figure S1) and included (1) an initial 20-min online survey (Survey 1, Summer 2023); (2) a 5-h online interactive Deliberative Workshop (Fall 2023); and (3) a 20-min online follow-up survey (Survey 2, Spring 2024). Delphi methods were modified and adapted from those of other studies with a similar design^45^^,^^46^^,^^47^ and specific methodology guidance.^48^^,^^49^^,^^50^^,^^51^ The study was organized around two primary objectives pertaining to research domains: (1) the identification of key characteristics for implementing wGT, and (2) an evaluation of stakeholder acceptance of wGT. This study was determined to be exempt from institutional review board (IRB) oversight by the IRBs of The Jackson Laboratory, The University of Michigan, The University of Iowa, and MaineGeneral Medical Center. Additional details about the methods used for the modified Delphi process and an overview of the process (Figure S1) can be found in the supplemental materials. Participants were required to have completed each prior step in order to continue participating in the study.

The research team sent eligibility screeners to 139 potential stakeholders, including employees, employers, ELSI professionals, healthcare professionals, and genetic testing industry representatives. This approach yielded *n* = 96 screened eligible potential stakeholders for research participation. From this list, the study team selected *n* = 53 potential stakeholders to send the study consent and Survey 1, prioritizing diversity in types of stakeholder perspectives and demographic characteristics. The goal was to recruit approximately 8–10 participants in each stakeholder group, keeping the groups comparable in size.

### Identifying key characteristics for implementation of wGT

Many of the items in Survey 1 were designed to elicit participants’ initial perspectives on the potential benefits, harms, and programmatic design of wGT to provide the groundwork for future group discussions about potential key characteristics for wGT during the Deliberative Workshop. Survey 1 included free-text response items in which participants were asked to independently list and rank up to five (1) potential benefits of wGT, (2) potential harms of wGT, and (3) potential design features important to consider for wGT. The research team developed a codebook inductively based on participants’ responses to Survey 1 as well as the principles of thematic analysis. Benefits, harms, and design features of wGT were thematically coded. The aggregated responses were analyzed to identify the most frequent themes among participants’ responses.

During the Deliberative Workshop, two research team members (W.G.F. and A.E.R.P.) provided educational presentations on workplace wellness programs, wGT, and relevant laws (e.g., GINA) and shared the aggregate, deidentified results of Survey 1 with participants, including the top ranked benefits, harms, and design features important to consider for wGT. Participants were assigned to one of five small groups, broadly segregated by stakeholder groups (employee, employer, ELSI scholar, healthcare provider, genetic testing industry representative). Small group discussions focused on benefits, harms, and design features for wGT. Given the inclusion of stakeholders with diverse experiences and expertise, brief post-workshop evaluation was developed based on content from other studies.^46^^,^^47^^,^^52^ The post-workshop evaluation was administered immediately after the Deliberative Workshop to assess stakeholder satisfaction and comfort level during the workshop, perceptions of bias from the research team, and group dynamics. Audio recordings of the Deliberative Workshop were transcribed verbatim and deidentified by Landmark Associates. Research team members created short qualitative summaries for each transcript. These summaries were reviewed with attention to recommendations for the design and implementation of wGT to maximize benefits and minimize harms. From this information, 12 key characteristics were identified by the research team and representative quotes were collected for each key characteristic by a qualitative researcher (K.A.R.).

In Survey 2, participants were presented with each of the 12 key characteristics in a random order and asked to rate their agreement with the (1) importance, (2) likelihood of being achieved (Likert scale: 1 = *Strongly agree*, 5 = *Strongly disagree* for importance and likelihood of being achieved), and (3) necessity in order for wGT to be offered (1 = *Yes, I agree*, 2 = *No, I disagree*) for each characteristic. These data were summarized with descriptive statistics.

### Evaluating stakeholder acceptance of wGT

We evaluated stakeholder perspectives on acceptance of wGT (e.g., whether it should be offered by employers, or allowed to exist altogether), and how their perspectives changed over the course of the modified Delphi process. Likert-scale questions were administered at multiple stages of the process: During Survey 1 and at the beginning of Survey 2, participants were asked to rate their agreement on a 5-point Likert-type scale (1 = *Strongly agree*, 5 = *Strongly disagree*) with two statements deliberately designed to be dichotomizing regarding acceptance of wGT: (Q1) *Employers should NOT be allowed to offer wGT*; (Q2) *Employers should offer wGT*. During the Deliberative Workshop, one plenary session was dedicated to discussion on these questions about acceptance of wGT. Data on Q1 and Q2 were presented in an aggregate, deidentified form to participants during the Deliberative Workshop and in Survey 2. At the end of Survey 2, participants were asked to respond to these two questions again, this time, assuming hypothetically that the key characteristics they previously indicated were “necessary for wGT to be offered” had been met.

We undertook several types of analyses to analyze participant responses to the items assessing stakeholder acceptance of wGT over the course of the modified Delphi process. Only those participants who completed all three rounds of the process were included in these analyses. First, we inductively created five descriptive categories of responses to these items: (1) Supportive (disagreed on the Likert scale that employers *should not be allowed to offer* and agreed that employers *should offer* wGT), (2) permissive (disagreed that employers *should not be allowed to offer* and neutral that employers *should offer* wGT), (3) neutral/conflicted (neutral on whether employers *should be allowed to offer* wGT), (4) libertarian (disagreed that employers *should not be allowed to offer*, and disagreed that they *should offer* wGT), and (5) opposed (agreed that employers *should not be allowed to offer* and disagreed that they *should offer* wGT) (Table 1). The descriptive categories pertaining to participants’ acceptance of wGT were reviewed for both participants who dropped out of the study after Survey 1 and those who completed the entire study.

**Table 1:** Categories of stakeholder acceptance of workplace genetic testing (wGT) and associated response criteria

Category	Response criteria^a^	
	Q1. Employers should offer wGT	Q2. Employers should NOT be allowed to offer wGT
Supportive	somewhat agree or strongly agree	somewhat disagree or strongly disagree
Permissive	neither agree nor disagree	somewhat disagree or strongly disagree
Neutral	neither agree nor disagree, somewhat disagree, or strongly disagree	neither agree nor disagree
Libertarian	somewhat disagree or strongly disagree	somewhat disagree or strongly disagree
Opposed	somewhat disagree or strongly disagree	somewhat agree or strongly agree

a Likert-scale answer choices for Q1 and Q2 included the following: Strongly agree, somewhat agree, neither agree nor disagree, somewhat disagree, strongly disagree.

Categories were assigned to participants’ responses by comparing quantitative Likert-type scale scores to qualitative open-ended responses, highlighted by representative quotes. Next, we assessed the extent to which participants changed their opinions throughout the modified Delphi process. Chi-square analysis was used to determine if there were significant differences in participants’ likelihood of moving toward agreement or disagreement with these two statements at each step of the modified Delphi process.

## Results

### Study participants

A total of 43 participants consented and completed Survey 1 (Tables 2 and S1). The majority were White (*n* = 31 of 43, 72%), female (*n* = 27 of 43, 63%), and had a postgraduate or professional degree (*n* = 36 of 43, 84%) (Table 2). Thirty-one participants attended the Deliberative Workshop, and 30 participants completed Survey 2. The highest attrition was observed in the healthcare professional and employer stakeholder groups between Survey 1 and the Deliberative Workshop (Table S1). Anecdotally, the reasons most often given for dropping out in participants’ communications to study investigators related to conflicting time commitments. Overall, the percentage of participants who began the study and indicated they were supportive or permissive of wGT on Survey 1, but did not complete the entire study, mirrored that of those who completed the entire study. Results of the post-workshop evaluation indicated that most participants either somewhat or strongly agreed that (1) they were satisfied with their experience (*n* = 26 of 29, 90%), (2) their viewpoint was taken seriously (*n* = 27 of 29, 93%), (3) they felt comfortable participating (*n* = 27 of 29, 93%), and (4) the research team presenters were unbiased on the topic (*n* = 27of 29, 93%) (Table S3).

**Table 2:** Survey 1 participant characteristics (*N* = 43)

Participant variable	*n* (%)
**Primary stakeholder group**^a^	

Employee	8 (19)
Employer	8 (19)
ELSI professional	10 (23)
Genetic testing company/industry representative	8 (19)
Healthcare professional	9 (21)

**Gender**	

Female	27 (63)
Male	15 (35)
Non-Binary	1 (2)

**Race and ethnicity**	

Asian	2 (5)
Black, non-Hispanic	2 (5)
Hispanic/Latino	5 (12)
White, non-Hispanic	31 (72)
More than one race and ethnicity	3 (7)

**Age, y**	

25–35	9 (21)
36–45	6 (14)
46–55	13 (30)
56–65	7 (16)
≥66	8 (19)

**Education**	

Postgraduate or professional degree	36 (84)
Four-year college or university degree	6 (14)
Some graduate school	1 (2)

a Multiple participants identified with more than one stakeholder group. The research team categorized employees into stakeholder groups based on publicly available information about each individual, which sometimes did not match the self-reported stakeholder group (e.g., some healthcare professionals selected “employee,” but their professional healthcare experience was prioritized for this study).

### Identifying key characteristics for implementation of wGT

During the Deliberative Workshop, participants were shown, in aggregate, their self-identified perceived benefits and harms of wGT from Survey 1. Top potential benefits of wGT raised by participants in Survey 1 included positive health outcomes, knowledge about genetic risk, and access to genetic testing. Top potential harms included privacy-related harms, discrimination, lack of understanding, negative emotions, and lack of access to follow-up care (full descriptions can be found in Table S2). During the Deliberative Workshop discussions of these findings, 12 key characteristics of benefit-maximizing and harm-reducing wGT programs emerged (Table 3). Small groups tended to focus on slightly different aspects or details of wGT in their Deliberative Workshop discussions; however, all of the groups endorsed and discussed in detail both the potential harms and potential benefits of wGT. We did not observe any overt differences among small group discussions in relationship to the identification of key characteristics for implementing wGT, or their overall assessment of wGT. Furthermore, when small group discussions were brought back and reported out to the larger group, additional deliberation tended to reveal commonalities, as opposed to divergences, in discussions.

**Table 3:** Twelve key characteristics identified through the modified Delphi process to maximize the potential benefits of workplace genetic testing (wGT) while minimizing potential harms

Key characteristic	Description: Measures to ensure …	Representative quote(s)
Anti-discrimination	… prevention of unfair treatment based on data from wGT.	ELSI stakeholder: *I would think that one of the biggest risks is what happens to that information and then how can it be used—essentially, how can it be used against me? Then not only me, but actually my family, and how does it impact my insurance rates? How does it impact my employability? Recognizing that there are laws that prevent discrimination, we also acknowledge that there are ways and loopholes that happen with laws all the time. For me, there would be a lot of fear or concern about having this information available to my employer … It’s the fear of unknown and not knowing the details of the law and how to ensure that my employer sticks to it without having to get lawyers and get involved … It’s a fear of, what do you do when something goes awry … it’s a lot to have to take on and to monitor.*
Employee control	… employees are in charge of the management and use of data from wGT.	Employer stakeholder: *It is about the employee data control slash EMR integration … making sure that [the] employee has some way to get their data. They own it. They control it. Ideally, they can have access to the app in perpetuity.* ELSI stakeholder: *I was just wondering if there would be the options of ability in how you would transfer information potentially to transition that into the clinical space 'cause a lot of—if it comes in as a—I know, when it comes to clinical testing, we have just PDFs, not very useful over the long term, but there are health systems that have more sustained decision support that’s useful over the long term.*
Equity	… there is equitable access to wGT, related benefits, and follow-up.	Genetic testing industry stakeholder: *Going back to the disparities in terms of healthcare delivery. I think one big risk is that they get a result and then there’s no bridge to any sort of care and particularly when—especially with bigger companies, there’s wide variability in what health insurance plan somebody may have … if there’s no confidence that if you come back with a BRCA1 mutation that you can go and get yourself a breast MRI for a reasonable price, or that you could get your ovaries removed, if you so choose … Those are things that will have the potential to cause a lot more harm for those individuals, once they try to get to the next step … A lot of people can fall off, and then that information no longer becomes useful.* Employee stakeholder: *If you are offering a service where people can get genetically tested or whatever-not, are these employers, are they also going to offer, I don’t know, a week’s worth of, I don’t know, or 3 days' worth of PTO around that so that maybe you can then make the follow-up visits to a doctor … ?*
Evidence-based testing	… testing is performed by a reputable laboratory and that what is analyzed on the test is supported by evidence and produces accurate results.	Genetic testing industry stakeholder: *I wanna see actionable evidence-based testing from a [Clinical Laboratory Improvement Amendments and College of American Pathologists (CLIA/CAP)-certified laboratory]. It falls under clinical laboratory testing rules and regulations.* Employer stakeholder: *Yeah, put in a pitch for evidence-based, I think. That’s because that’s of big importance to me. Tests where you don’t have a sufficient evidence base to understand the upsides and the downsides and how those might be balanced, I think, just makes for bad testing … There’s a lot of information freely available in the literature and online about what … you might put together for a panel. You might have to delve in a little deeper to understand whether or not there’s any … utility actually to using that panel over current clinical practice. Making sure that thought goes into what tests are included and how it’s reported out. I think for me evidence-based is an essential item.* Employee stakeholder: *I have one … accuracy. What is the accuracy of these tests? Are we talkin' 80%, 99%? What? Because if the results are not 100%, or close to it, accurate, then they’re pretty worthless.*
Healthcare integration	… that wGT results can be integrated into the healthcare system and/or the electronic medical record.	Genetic testing industry stakeholder: *Then the lack of access to follow-up I think is really a serious problem. There’s no, if—if I should say—if there is no clear path to having had an employee wellness test, if something comes up that is of significance, there’s a way to immediately get into counseling and follow-up care.* Employee stakeholder: *Yeah, no, and there is that disconnect, right now, between this—these results and your normal health insurance because you may wanna have a follow-on test, but you need to then pass that along to your health insurance. I don’t know. I don’t think that happens through the employer 'cause they don’t really—I mean, they’re—the health insurance is a separate entity. It may be direct with the—I mean, I don’t know. Maybe you have to sign a release of information to send it to your health insurance. I don’t know, but I don’t think it would be the employer.*
Privacy/security	… the confidentiality and security of wGT results.	Healthcare professional stakeholder: *… employers should not be able to get their hands on individualized data about their employees. That’s actually in the laws. Still, that would be a prerequisite.* Employee stakeholder: *I think we spoke a lot about, I think—what was it, breach of data? I think it was <other participant> who said that, and scared me, that it's hard to code things as private once you get that with a lot of data or participant information. I don’t know. Going off of that, I would think privacy-related features, that makes sense if we’re gonna combat breach of data.*
Transparency	… there is clear communication about how the wGT program works—the process and data protections.	ELSI stakeholder: *The employees are not gonna see the terms of that contract. They’re not gonna know what the data agreements necessarily are and the details about the stated purposes of those data. There might be something that’s—like a notice that’s given to people, the employees who might be participating there that says, "We’re gonna aggregate data. Any data would be aggregated or deidentified and shared with the employer and won’t be used against you," or something like that.* Employee stakeholder: *I think information on what’s involved … the information on what GINA is all about and how it affects the use of my data would at least make me feel better about the genetic testing.*
Understanding/education	… employees have a good understanding of wGT before and after the test (for example, education, genetic counseling).	Genetic testing industry stakeholder: *I’ll just say I support access to [wGT]. I support employers wanting to institute these programs as long as they have the right educational programs in place so that patients understand the kinds of information that they’re getting, and what they may or may not be able to do with that information.* ELSI stakeholder: *I think that there needs to be good education around whatever is being offered. I think it’s also hard because most of this is sort of in a direct-to-consumer way. I’m assuming they don’t always require genetic counseling … probably because it would make it more expensive. Especially for people who are not genetics experts taking these tests, there could be misunderstanding of what the information is telling them or false reassurance if they have a negative result or nothing is identified. I think there’s a danger in misunderstanding and then feeling safe from whatever it is that you didn’t have … I think that is definitely a risk that would be hard to get around. 'Cause even if you’re providing the education, people don’t always pay attention to that. I skip a lot of stuff that I should be reading and just, "Okay. That looks good. I’ll sign it." I think that’s a risk.*
User-friendliness	… the wGT program has a simple and easy-to-use design.	Healthcare professional stakeholder: *In addition to the option to have the in-person consultative telegenetics type of consultative service offering information in different formats and modalities … it’d be great to have a video somebody could just click and see and get some information. Just understanding people getting information in different ways or receive that information in different ways. They may not have time to sit down and have a counseling session. Maybe they can listen to a video in their car while they’re riding.* Employee stakeholder: *I don’t know if this falls under user friendliness or not, but how is the test actually administered? Because I think it’s one thing if it’s just you put a piece of your hair in an envelope and you mail it back in … Or is it something where you have to do a blood draw or a mouth swab? If so, is your workplace gonna offer that test at the workplace? Are you gonna be given release time to go and get this done? Are you expected to do it on your own time?*
Utility for employers	… employers can reap benefits through offering wGT to their employees.	ELSI stakeholder: *Employees may appreciate the idea that their employer cares about them and their health and thinks—people like getting, quote, perks. To the extent that people feel better about their employer and their employment as a result of having this option, that could be a benefit to employees as well as employers.* Employee stakeholder: *Maybe the assumption is that … [with wGT], maybe you’re gonna miss less sick leaves or PTO or what-not and work on, I don’t know, a lot of preventative measures and be at work and be more productive, and your mood is better. I don’t know. Maybe for the long run, maybe that’ll reduce employee costs, and they’ll also be alive. It’s one less person to hire.* Genetic testing industry stakeholder: *… the primary benefit for the employers is … to be able to appear to be very progressive and concerned and cutting edge, and to attract certain employers who may be of a particular mindset … they’re using it primarily as a competitive advantage …*
Utility for laboratories	… laboratories can reap the benefits of performing and/or offering services related to wGT.	Healthcare professional stakeholder: *For me, it’s a free market system. Vendors are seeing a market and an opportunity … to make a business plan or a business model out of offering [wGT].* ELSI stakeholder: *It’s in [vendors’] interest to emphasize the purported benefits of testing of all sorts, and I would assume that genetic testing would fall into that broad category.*
Voluntariness	… prevention of coercion or pressure to participate in wGT.	Genetic testing industry stakeholder: *I wanted to touch on the voluntariness, lack of coercion. To me that is not a design feature, that’s a requirement … if you can’t even offer it without coercing individuals, then … there’s no way to ever offer it.* Employee stakeholder: *I, also, would make voluntariness or lack of coercion a must-have. Requiring genetic testing is a little much for me. It’s a little 1984 or somethin' like that. Think it has to be voluntary.*

**Figure 1:**
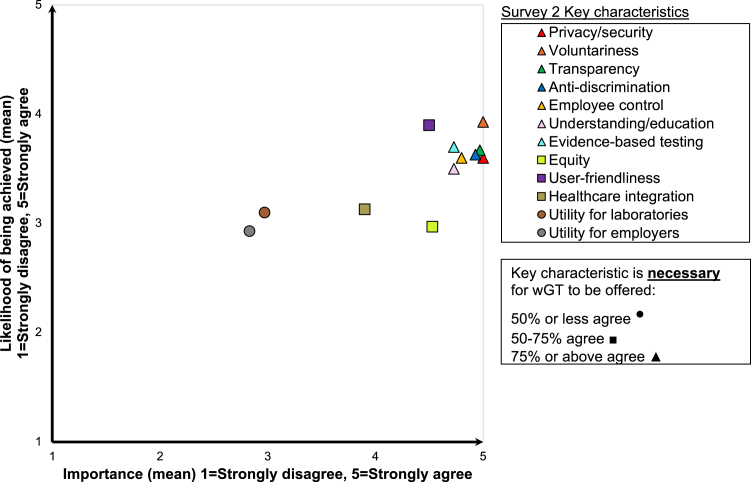
Key characteristics of workplace genetic testing (wGT) and their importance, necessity, and likelihood of being achieved as perceived by participants (*n* = 30) Importance (*x*), likelihood of being achieved (*y*), with shapes of symbols (legend) denoting the percentage of participants who indicated the key characteristic was “necessary” for wGT to be offered. Values plotted on the *x*/*y* axes represent mean of Likert-scale scores for each characteristic.

While most participants agreed that privacy/security, voluntariness, transparency, understanding/education, anti-discrimination, employee control, and evidence-based testing were important (>90%) and necessary (>75%) for implementation of wGT, some expressed doubts about their likelihood of being achieved (Figures 1 and S4). Equity and healthcare integration were considered relatively important but were also seen as less likely to be achieved than the other key characteristics. In addition, utility for laboratories and utility for employers received low mean scores on both importance and likelihood of being achieved (Figure 1). Figures S2–S4 provide additional details about participant endorsement of importance, likelihood of being achieved, and necessity of the 12 key characteristics for wGT.

### Evaluating stakeholder acceptance of wGT

The majority of participants were categorized as either supportive (*n* = 10 of 30, 33%) or libertarian (*n* = 8 of 30, 27%) regarding wGT based on their Likert-scale responses on Survey 1 (Table 4). By Survey 2, after the Deliberative Workshop, most participants had shifted to either permissive (*n* = 12 of 30, 40%) or opposed (*n* = 8 of 30, 27%) to wGT, indicating increased skepticism. In Survey 2, when assuming that the key characteristics they deemed “necessary” for wGT to be offered were achieved, most participants were either categorized as supportive (*n* = 11 of 30, 37%) or permissive (*n* = 9 of 30, 30%) of wGT (Table 4). While it appeared that individuals in the employee and genetic testing industry stakeholder groups began the study slightly more supportive or permissive than others, major differences in acceptance of wGT by stakeholder group were not clearly observed, both in Survey 1 and throughout the study.

**Table 4:** Shifts in stakeholder acceptance of workplace genetic testing (wGT) before and after the deliberative workshop based on quantitative and open-ended survey responses (*n* = 30)

Category^a^	Survey 1^b^*N* (%)	Survey 2^b^*N* (%)	Survey 2, assuming key characteristics met^b^*N* (%)	Exemplary quote
Supportive	10 (33)	4 (13)	11 (37)	*Given the types of benefits currently being offered by employers, I think genetic testing fits in well and is a compelling benefit, especially as genetic testing accessibility is so limited and is not being provided even to those who meet criteria and should be getting it. This provides the opportunity for access to more people.*
Permissive	4 (13)	12 (40)	9 (30)	*I believe that if the employer determines that there is substantial value to the employee and, by extension, to the organization in offering the testing, the employer should be allowed to do so. I do not believe that employers have an obligation to offer this service, but neither do I feel that they should be discouraged from doing so. In essence, I find no absolute imperative to pursue either course of action.*
Neutral/Conflicted	6 (20)	3 (10)	1 (3)	*It depends on the safeguards in place.*
Libertarian	8 (27)	3 (10)	5 (17)	*I can’t support any measure to prevent what an employer offers as an employee option. The marketplace should determine an employer’s benefit programs.*
Opposed	2 (7)	8 (27)	4 (13)	*… I believe the workplace should keep their grubby, greedy hands off of my genetics.*

a Participants perspectives on whether wGT should (1) be offered, and (2) not be allowed, from Survey 1, to Survey 2, to Survey 2 assuming the 12 key characteristics are met. Categories based on levels of support or opposition to wGT were assigned to groups of participants based on quantitative survey responses and corresponding exemplary quotes (supportive, permissive, neutral/conflicted, libertarian, or opposed; see methods, Table 1 for additional details).

b The Deliberative Workshop took place between Survey 1 and Survey 2.

Quantitative analysis supported descriptive trends. Results of chi-square analysis revealed participants were significantly more likely to move toward agreement that wGT should NOT be allowed after the Deliberative Workshop, between Survey 1 and Survey 2 (*p* = 0.02). However, from the beginning to the end of Survey 2, participants were significantly more likely to move toward (1) disagreement that wGT should NOT be allowed (*p* = 0.02) and (2) agreement that wGT should be offered (*p* = 0.01), assuming their key characteristics were met (Table 5).

**Table 5:** Shifts in stakeholder acceptance of workplace genetic testing (wGT) before and after the Deliberative Workshop based on quantitative survey responses and chi-square analysis (*n* = 30)

Survey question^a^ (timing of survey)	No change in agreement *N* (%)	Shifted toward disagree*N* (%)	Shifted toward agree*N* (%)	*P* value^b^
**Q1. Employers should NOT be allowed to offer wGT**^c^				

(Survey 1 vs. Survey 2)	16 (53)	3 (10)	11 (37)	0.02
(Survey 1 vs. Survey 2, Assuming key characteristics met)	16 (53)	7 (23)	7 (23)	1.0
(Survey 2 vs. Survey 2, Assuming key characteristics met)	22 (73)	7 (23)	1 (3)	0.02

**Q2. Employers should offer wGT**^c^				

(Survey 1 vs. Survey 2)	15 (50)	10 (33)	5 (17)	0.14
(Survey 1 vs. Survey 2, Assuming key characteristics met)	14 (47)	7 (23)	9 (30)	0.56
(Survey 2 vs. Survey 2, Assuming key characteristics met)	18 (60)	2 (7)	10 (33)	0.01

a Participants were asked to rate their agreement with Q1 and Q2 in both Survey 1 and Survey 2. The Deliberative Workshop took place between Survey 1 and Survey 2. Later in Survey 2, participants were again asked to rate their agreement with these items, assuming the key characteristics for wGT programs that they described as “necessary” were met.

b Chi-square analysis was used to compare the percentage of participants who shifted toward *disagreement* with the percentage who shifted toward *agreement* with Q1 and Q2 before and after the Deliberative Workshop.

c Likert-scale question: Strongly agree to strongly disagree.

## Discussion

We conducted a modified Delphi process with workplace genetic testing stakeholders to identify and assess key characteristics for implementation of wGT that could maximize benefits and minimize harms, and to explore stakeholder acceptance. Twelve key characteristics emerged: employee control of data, equity in access, evidence-based testing, healthcare integration, programmatic transparency, user-friendliness, utility for employers, utility for laboratories, pre- and post-test education, voluntariness of participation, anti-discrimination measures, and the privacy and security of information (Table 3). The latter four mirror many of the top ethical, legal, and social criteria identified in previous studies on dtcGT.^42^^,^^43^^,^^44^ The identified key characteristics also align well with accepted ethical principles in clinical care delivery,^53^ research ethics,^54^ and public health practice.^55^ Programmatic design features that supported employee data control were reported to increase employee participation in a hypothetical wGT program in one recent study,^16^ which is also consistent with our findings. While many of these key characteristics are not unique to wGT, and may broadly apply to genetic and genomic testing, the implications for achieving them require careful consideration of the employee-employer relationship. For example, in order for privacy and security of genomic data to be achieved in a wGT program, one must consider the employer’s potential access to data and the relevant laws, as GINA currently explicitly allows employers to receive aggregate employee data on wGT.^6^^,^^56^

Most participants agreed that these key characteristics were important and necessary for implementation of wGT. However, some participants expressed doubts about the likelihood of achieving all key characteristics due to structural challenges, such as ensuring equitable access to genetic and healthcare services for all employees, incorporating genetic testing data into electronic health record systems, and providing adequate genomics education for healthcare providers. These findings raise questions about the feasibility of implementing wGT in adherence to stakeholders’ ethical values and preferences. Implementation studies may help to further clarify the feasibility of incorporating such elements in wGT program design.

These findings may also point to an overall, underlying lack of trust in employers and genetic testing vendors in general to ensure employee privacy, security, and nondiscrimination. Prior to the introduction of GINA, the potential for overt genetic discrimination in the workplace was a concern. For example, in *Norman-Bloodsaw v. Lawrence Berkeley Labs* (1998), an employer was sued for testing Black employees for sickle cell carrier status without their knowledge.^6^^,^^57^ Although GINA now provides some genetic privacy protections, including protections against genetic discrimination in employment, studies have shown that the public lacks awareness of GINA, and its existence does not fully alleviate concerns about potential genetic discrimination.^10^^,^^32^^,^^58^ Other recent events, such as 23andMe’s data breach and bankruptcy announcement have shed light onto the challenges of trusting private companies, outside the healthcare arena, with sensitive genetic data.^59^^,^^60^^,^^61^

Overall, participants’ responses to questions measuring their acceptance of wGT demonstrated they had qualified support for wGT being offered, conditional on whether all characteristics that individuals felt were “necessary” could be met. These findings may suggest that wGT could be a viable model for offering genetic testing. However, ensuring that key characteristics are achieved would require substantial policy development and significant changes to infrastructure and processes within companies, genetic testing laboratories, regulatory bodies, and healthcare systems. For example, since stakeholders expressed concerns about the feasibility of integrating wGT into existing healthcare systems, it would be necessary to develop effective methods for ensuring that genetic test results from wGT can be easily and securely shared with healthcare providers via electronic health records, with employees’ consent. Additionally, given employee data control was deemed very important by most stakeholders, it might be important to consider how employees would be able to withdraw their wGT-related data, including deidentified data from a laboratory’s database, which may require organizational policy and legal changes related to laboratory record retention. These key characteristics should be considered as workplace genetic testing continues to be implemented,^62^ and as workplace wellness programs involving genetic testing evolve.^63^

### Strengths and limitations

Our study is one of the first to systematically define key characteristics for wGT that, if achieved, could maximize potential benefits while reducing risks of harm. A strength of the study is that the findings are derived from a diverse group of stakeholders, including those who are potentially involved in or directly affected by wGT. The design of the modified Delphi process combined with a Deliberative Workshop ensured that stakeholders of varying levels of genetic expertise could have their perspectives heard and validated in the development of the key characteristics. However, the generalizability of our findings is likely limited by the relatively small number of participants from each stakeholder group. Furthermore, despite our efforts to recruit for diversity, the diversity within our study population was not as high as desired. Additional national studies investigating stakeholder perspectives^9^^,^^10^ are needed to develop a more comprehensive understanding of wGT stakeholder viewpoints.

We also observed a 30% attrition rate, primarily among healthcare professionals and employers, while 100% of genetic testing industry representatives finished all steps of the study, possibly biasing our results toward the opinions of this group. However, the attrition rate was relatively small compared with other multi-step Delphi processes that primarily include surveys.^64^ Our modified Delphi process required more of participants, who were asked to engage in a 5-h deliberative workshop.

We specifically chose dichotomous questions to evaluate stakeholder acceptance of wGT, which included one potentially confusing negative statement (e.g., “Employers should NOT be allowed”). We observed apparent contradictions in how participants answered these questions (e.g., three quantitative survey responses did not seem to match the associated qualitative response) and received feedback from participants during the workshop that the dichotomous format was challenging. Nevertheless, framing the questions as a dichotomous choice allowed us to track changes in participants’ perspectives over time and to determine whether the key characteristics would influence their ultimate acceptance of wGT.

### Conclusions

This study presents the results of the first modified Delphi process to explore important stakeholder perspectives in order to identify key characteristics for maximizing benefit and reducing potential harm in the implementation of wGT. The characteristics prioritized by stakeholders should be considered in the programmatic design of wGT. However, given stakeholders’ lack of confidence in achieving some of the proposed key characteristics in the context of wGT, further evaluation of their feasibility is needed. These findings help to inform the development of a normative framework for wGT.

## Data and code availability

The survey instruments are available in the supplemental materials of this article. Other study materials and quantitative data that support the findings of this study are available at Inter-university Consortium for Political and Social Research (ICPSR) based at the University of Michigan under ICPSR-229864. The raw qualitative data are not publicly available due to privacy or ethical restrictions.

## Consortia

The INSIGHT @ Work consortium includes the following core team members, in addition to the named authors: Betty Cohn, MBE, Nicole Crumpler, MS, MBA, Rebecca Ferber, MPH, Veda N. Giri, MD, Katherine Hendy, MA, PhD, Amy Leader, MPH, DrPH, Debra Mathews, MA, PhD, Sarah McCain, MPH, Kayte Spector-Bagdady, MBE, JD, and Wendy R. Uhlmann, MS, CGC. The consortium is supported by advisory board members Kyle Brothers, Ellen Wright Clayton, Patricia Deverka, Thomas Ellis, Aaron Goldenberg, Susan Mockus, Cynthia Casson Morton, Jens Rueter, and Brett Witham, along with stakeholder workgroup members Ethan Bessey, Erynn Gordon, LaTasha Lee, Jessica Roberts, and Fatima Saidi

## Acknowledgments

This research was supported by a grant from the 10.13039/100000051National Human Genome Research Institute of the 10.13039/100000002National Institutes of Health (R01HG010679). The content is solely the responsibility of the authors and does not necessarily represent the official views of the National Institutes of Health.

## Author contributions

First author E.C. led the writing of the original draft, curated all data, and contributed to formal analysis, methodology, investigation, and project administration with supervision from K.S. Senior authors A.E.R.P. and W.G.F. led the investigation and methodology, provided supervision, and contributed to formal analysis and project administration. E.C., K.S., K.A.R., A.V., A.T., A.E.R.P., and W.G.F. contributed to conceptualization, investigation, data curation, and project administration. K.A.R. provided additional expertise regarding methodology and contributed to formal analysis. K.S., K.A.R., A.T., A.E.R.P., and W.G.F. also contributed to the original draft and provided supervision during the writing process. S.P. and J.M.R. provided software and conducted inferential statistical analysis. K.S., C.L., and J.S.R. were responsible for funding acquisition. C.L. and J.S.R. provided oversight of all research and administrative activities as multiple principal investigators. All authors read and approved the final manuscript.

## Declaration of interests

The authors declare no competing interests.

## Supplemental information

Document S1. Figures S1–S4 and Tables S1–S3

Document S2. Article plus supplemental information

## Footnotes

Supplemental information can be found online at https://doi.org/10.1016/j.xhgg.2025.100458.
