# Supplementary material for: Multidisciplinary stakeholder-informed identification of key characteristics for implementation of workplace genetic testing
Source: HGG Adv. 2025 May 22;6(3):100458. doi: 10.1016/j.xhgg.2025.100458 (PMC12169765; doi:10.1016/j.xhgg.2025.100458)
Supplement: Document S1. Figures S1–S4 and Tables S1–S3 [file mmc1.pdf]

HGGA, Volume *6*

Supplemental information

Multidisciplinary stakeholder-informed identification of key characteristics for implementation of workplace genetic testing

Elizabeth Charnysh, Kunal Sanghavi, Kerry A. Ryan, Alyx Vogle, Alexandra Truhlar, Subhamoy Pal, Jonathan M. Reader, J. Scott Roberts, Charles Lee, Anya E.R. Prince, W. Gregory Feero, and INSIGHT @ Work Consortium

Supplemental Materials

SURVEY INSTRUMENTS

SURVEY 1

Demographics Please fill out the following demographic characteristics so we may appropriately understand your role as stakeholder in this deliberative process. **Click the arrow below to continue.**

Start of Block: Demographics

What are your stakeholder role(s)/perspectives with regard to the genetic testing wellness programs? Please select your **primary perspective** for this process.

- ○ Employee organization (e.g., union leader, employee advocacy group)
- ○ Employer or human resources
- ○ Ethical, legal, and social implications or bioethics
- ○ Genetic counselor or other healthcare professional
- ○ Genetic testing company
- ○ Government regulator (e.g., EEOC, HHS)
- ○ Insurance company
- ○ Privacy and/or data protection
- ○ Employee
- ○ Other (please specify): ____

Please briefly describe your current or most recent role in this field.

____

____

____

____

____

How many years have you been in this field?

- Less than 1 year
- 1-4 years
- 5-10 years
- 11-15 years
- More than 15 years

Page Break

What is your highest level of education?

- Some high school
- High school graduate (diploma or GED certificate)
- Some college, no degree (includes some community college)
- Two-year associate degree from a college or university
- Technical/trade school or apprenticeship (e.g., electrician, plumbing, culinary school)
- Four-year college or university degree/Bachelor’s degree (e.g., BS, BA, AB)
- Some postgraduate or professional schooling, no postgraduate degree (e.g., some graduate school)
- Postgraduate or professional degree, including master's, doctorate, medical or law degree (e.g., MA, MS, PhD, JD, graduate school)

What is your age?

____

End of Block: Demographics

Start of Block: Delphi Round 1 Intro

Delphi Survey: Round 1
As part of a workplace wellness program, a number of US employers are offering their employees workplace genetic testing (wGT). We are convening a deliberative process including a workshop with key stakeholders (including employees, employers, labor organization representatives, ethicists, insurance companies, genetic testing companies, health care providers, policy makers and legal experts) to identify the ethical, legal, social, and policy implications of wGT and to suggest practice and policy options to guide implementation and governance of such testing. Please complete this 20 minute survey that will help inform the workshop. **Click the arrow below to continue.**

End of Block: Delphi Round 1 Intro

Start of Block: Block 9

Please watch the less-than-5-minute video below before proceeding to learn important background information about the research study.

The survey includes a short video. You will need to play it with sound. If you are not able to have your sound on at this time, feel free to take this survey later when you can play the sound. If you will not be able to hear the video, click on the CC button to enable closed captioning.

https://youtu.be/kyTCELnP3Bs

End of Block: Block 9

Start of Block: Survey Part 1

Perspectives on workplace genetic testing Please review the definition of workplace genetic testing before continuing.

Workplace genetic testing: Voluntary genetic testing offered to employees as part of a workplace wellness program. This testing is not initiated by a provider and is not typically discussed in a visit with their clinician prior to testing. For example, a company may offer their employees the option to pursue genetic testing for inherited conditions that increase someone's risk for cancer or heart disease.

For each of the next 11 questions, please describe your answers in detail and give examples as needed. There are no right or wrong answers; we want to know your experiences, views, and perceptions. You will be given an opportunity to provide possible benefits and risks of workplace wellness genetic testing.
Click the arrow below to continue.

End of Block: Survey Part 1

Start of Block: Benefits

Please describe **up to five** potential **benefits** of workplace wellness genetic testing.

○ ____

○ ____

○ ____

○ ____

○ ____

Workplace genetic testing: Voluntary genetic testing offered to employees as part of a workplace wellness program. This testing is not initiated by a provider and is not typically discussed in a visit with their clinician prior to testing. For example, a company may offer their employees the option to pursue genetic testing for inherited conditions that increase someone's risk for cancer or heart disease.

Carry Forward Entered Choices - Entered Text from "Please describe up to five potential benefits of workplace wellness genetic testing."

Please click and drag to rank **up to five** potential **benefits** of workplace wellness genetic testing.

______

______

______

______

______

Please describe any other potential **benefits** of workplace wellness genetic testing.

____

____

____

____

End of Block: Benefits

Start of Block: Risks/Harms

Please describe **up to five** potential **risks/harms** of workplace wellness genetic testing.

○ ____

○ ____

○ ____

○ ____

○ ____

Workplace genetic testing: Voluntary genetic testing offered to employees as part of a workplace wellness program. This testing is not initiated by a provider and is not typically discussed in a visit with their clinician prior to testing. For example, a company may offer their employees the option to pursue genetic testing for inherited conditions that increase someone's risk for cancer or heart disease.

Carry Forward Entered Choices - Entered Text from "Please describe up to five potential risks/harms of workplace wellness genetic testing. "

X→

Please click and drag to rank **up to five** potential **risks/harms** of workplace wellness genetic testing.

______

______

______

______

______

Please describe any other potential **risks/harms** of workplace wellness genetic testing.

____

____

____

End of Block: Risks/Harms

Start of Block: Design Features

In order to maximize the benefits you mentioned and minimize the risks you mentioned, what are the most important features to include when designing a workplace genetic testing program? Please describe **up to five** important **design features** to include for workplace wellness genetic testing.

○ ____

○ ____

○ ____

○ ____

○ ____

Carry Forward Entered Choices - Entered Text from "In order to maximize the benefits you mentioned and minimize the risks you mentioned, what are the most important features to include when designing a workplace genetic testing program? Please describe up to five important design features to include for workplace wellness genetic testing."

X→

Please click and drag to rank **up to five** important **design features** to include for workplace wellness genetic testing.

______

______

______

______

______

Please write any other important **design features** to include for a workplace genetic testing program.

____

____

____

____

____

Workplace genetic testing: Voluntary genetic testing offered to employees as part of a workplace wellness program. This testing is not initiated by a provider and is not typically discussed in a visit with their clinician prior to testing. For example, a company may offer their employees the option to pursue genetic testing for inherited conditions that increase someone's risk for cancer or heart disease.

End of Block: Design Features

Start of Block: Agree/Disagree and Why/Why Not?

Please indicate whether you agree or disagree with the following statement

	Strongly agree	Somewhat Agree	Neither agree nor disagree	Somewhat disagree	Strongly disagree
Employers **should not be allowed** to offer voluntary workplace genetic testing.	○	○	○	○	○

Please indicate whether you agree or disagree with the following statement

	Strongly agree	Somewhat Agree	Neither agree nor disagree	Somewhat disagree	Strongly disagree
Employers **should offer** voluntary workplace genetic testing.	○	○	○	○	○

Please expand upon your above responses.

____

____

Workplace genetic testing: Voluntary genetic testing offered to employees as part of a workplace wellness program. This testing is not initiated by a provider and is not typically discussed in a visit with their clinician prior to testing. For example, a company may offer their employees the option to pursue genetic testing for inherited conditions that increase someone's risk for cancer or heart disease.

POST-WORKSHOP EVALUATION

Thank you for participating in the workshop! Please indicate your level of agreement with the following statements. Then, click the arrow below to continue.

Overall, I was satisfied with the interactive workshop.

- ○ Strongly disagree
- ○ Somewhat disagree
- ○ Neither agree nor disagree
- ○ Somewhat agree
- ○ Strongly agree

My viewpoint was taken seriously during the workshop.

- ○ Strongly disagree
- ○ Somewhat disagree
- ○ Neither agree nor disagree
- ○ Somewhat agree
- ○ Strongly agree

My opinions were respected by my small group.

- Strongly disagree
- Somewhat disagree
- Neither agree nor disagree
- Somewhat agree
- Strongly agree

My perspectives and participation made an impact on the interactive workshop.

- Strongly disagree
- Somewhat disagree
- Neither agree nor disagree
- Somewhat agree
- Strongly agree

There were diverse stakeholder perspectives at the workshop.

- Strongly disagree
- Somewhat disagree
- Neither agree nor disagree
- Somewhat agree
- Strongly agree

My time participating in this workshop was worthwhile.

- Strongly disagree
- Somewhat disagree
- Neither agree nor disagree
- Somewhat agree
- Strongly agree

I felt comfortable participating in the interactive workshop.

- Strongly disagree
- Somewhat disagree
- Neither agree nor disagree
- Somewhat agree
- Strongly agree

The presenters were unbiased on the topic.

- Strongly disagree
- Somewhat disagree
- Neither agree nor disagree
- Somewhat agree
- Strongly agree

I would abide by the group's final position, even if it differs from my personal opinion.

- Strongly disagree
- Somewhat disagree
- Neither agree nor disagree
- Somewhat agree
- Strongly agree

I felt that my group had a good discussion, even if I personally held a different viewpoint than my other small group members.

- Strongly disagree
- Somewhat disagree
- Neither agree nor disagree
- Somewhat agree
- Strongly agree

Differences in power between the stakeholders **negatively affected** the conversation in my small group.

- Strongly disagree
- Somewhat disagree
- Neither agree nor disagree
- Somewhat agree
- Strongly agree

Do you have any comments you wish to share about the workshop or this study, in general?

____

____

____

____

____

SURVEY 2

Of note, questions asked that did not pertain to the findings of this paper have been removed from this copy of Survey 2.

Start of Block: Introduction

Introduction
Thank you for your participation in our assessment of **workplace genetic testing**. To date, you have completed the first survey and participated in a deliberative workshop to identify potential **benefits and harms** of workplace genetic testing programs, as well as **design features** that could possibly maximize potential benefits while minimizing potential harms. For a refresher on workplace genetic testing, please watch the 4-minute video on the next page.

Please select "next page" to continue.

Page Break

https://www.youtube.com/watch?v=kyTCELnP3Bs

Please select "next page" to continue.

End of Block: Introduction

Start of Block: Section I: Revisiting the Initial Questions

Section I: Revisiting the Initial Survey Questions
In the first survey round, we asked participants to rate their agreement with the following statements, and the group provided us with the following responses:

Please select "next page" to continue.

Page Break

X→ X→

Using the scales provided, please **share your current opinion** by rating your agreement with these statements:

	Strongly disagree	Somewhat disagree	Neither agree nor disagree	Somewhat agree	Strongly agree
Employers **should offer** workplace genetic testing.	○	○	○	○	○
Employers **should not be allowed** to offer workplace genetic testing.	○	○	○	○	○
Employers **should have the option** to offer workplace genetic testing.	○	○	○	○	○

Please select "next page" to continue.

Page Break

How have your thoughts about these questions changed since the first survey, if at all? Why or why not?

____

____

____

____

____

Please select "next page" to continue.

Page Break

End of Block: Section I: Revisiting the Initial Questions

Start of Block: Section II: Key Characteristics Intro

Section II: 12 Key Characteristics Based on the initial survey and the deliberative workshop, the participants of this study identified **12 key characteristics** that could potentially maximize benefits and minimize harms of workplace genetic testing.

Using the scales provided, **please rate your agreement** with the statements on the next pages about the importance, likelihood of being achieved, and necessity of the **12 key characteristics** for maximizing potential benefits and minimizing potential harms of workplace genetic testing programs.

As an individual participant in this study, you may or may not agree on the key characteristics identified by the study participants as a whole. In this second survey, we hope to gain a better understanding of where study participants **share common ground.**

Please select "next page" to continue.

End of Block: Section II: Key Characteristics Intro

Start of Block: Section II: Employee Control

Employee control: Measures to ensure employees are in charge of the management and use of data from workplace genetic testing.

	Strongly disagree	Somewhat disagree	Neither agree nor disagree	Somewhat agree	Strongly agree
Employee control is **important.**	○	○	○	○	○
Employee control is **likely to be achieved.**	○	○	○	○	○

Employee control is **necessary** in order for workplace genetic testing to be offered.

○ No, I disagree.

○ Yes, I agree.

Please select "next page" to continue.

End of Block: Section II: Employee Control

Start of Block: Section II: Transparency

Transparency: Measures to ensure there is clear communication about how the workplace genetic testing program works - the process and data protections.

	Strongly disagree	Somewhat disagree	Neither agree nor disagree	Somewhat agree	Strongly agree
Transparency is **important.**	○	○	○	○	○
Transparency is **likely to be achieved.**	○	○	○	○	○

Transparency is **necessary** in order for workplace genetic testing to be offered.

- No, I disagree.
- Yes, I agree.

Please select "next page" to continue.

End of Block: Section II: Transparency

Start of Block: Section II: Anti-Discrimination

Anti-discrimination: Measures to prevent unfair treatment based on data from workplace genetic testing.

	Strongly disagree	Somewhat disagree	Neither agree nor disagree	Somewhat agree	Strongly agree
Anti-discrimination is **important.**	○	○	○	○	○
Anti-discrimination is **likely to be achieved.**	○	○	○	○	○

Anti-discrimination is **necessary** in order for workplace genetic testing to be offered.

- No, I disagree.
- Yes, I agree.

Please select "next page" to continue.

End of Block: Section II: Anti-Discrimination

Start of Block: Section II: Privacy/Security

Privacy/security: Measures to ensure the confidentiality and security of workplace genetic test results.

	Strongly disagree	Somewhat disagree	Neither agree nor disagree	Somewhat agree	Strongly agree
Privacy/security is **important.**	○	○	○	○	○
Privacy/security is **likely to be achieved.**	○	○	○	○	○

Privacy/security is **necessary** in order for workplace genetic testing to be offered.

○ No, I disagree.

○ Yes, I agree.

Please select "next page" to continue.

End of Block: Section II: Privacy/Security

Start of Block: Section II: Voluntariness

Voluntariness: Measures to prevent coercion or pressure to participate in workplace genetic testing.

	Strongly disagree	Somewhat disagree	Neither agree nor disagree	Somewhat agree	Strongly agree
Voluntariness is **important.**	○	○	○	○	○
Voluntariness is **likely to be achieved.**	○	○	○	○	○

Voluntariness is **necessary** in order for workplace genetic testing to be offered.

○ No, I disagree.

○ Yes, I agree.

Please select "next page" to continue.

End of Block: Section II: Voluntariness

Start of Block: Section II: Understanding/Education

Understanding/education: Measures to ensure employees have a good understanding of workplace genetic testing before and after the test (for example: education, genetic counseling).

	Strongly disagree	Somewhat disagree	Neither agree nor disagree	Somewhat agree	Strongly agree
Understanding/education is **important.**	○	○	○	○	○
Understanding/education is **likely to be achieved.**	○	○	○	○	○

Understanding/education is **necessary** in order for workplace genetic testing to be offered.

- ○ No, I disagree.
- ○ Yes, I agree.

Please select "next page" to continue.

End of Block: Section II: Understanding/Education

Start of Block: Section II: Equity

Equity: Measures to ensure there is equitable access to workplace genetic testing, related benefits, and follow-up.

	Strongly disagree	Somewhat disagree	Neither agree nor disagree	Somewhat agree	Strongly agree
Equity is **important.**	○	○	○	○	○
Equity is **likely to be achieved.**	○	○	○	○	○

Equity is **necessary** in order for workplace genetic testing to be offered.

- ○ No, I disagree.
- ○ Yes, I agree.

Please select "next page" to continue.

End of Block: Section II: Equity

Start of Block: Section II: EBT

X→

Evidence-based testing: Measures to ensure testing is performed by a reputable laboratory and that what is analyzed on the test is supported by evidence and produces accurate results.

	Strongly disagree	Somewhat disagree	Neither agree nor disagree	Somewhat agree	Strongly agree
Evidence-based testing is **important.**	○	○	○	○	○
Evidence-based testing is **likely to be achieved.**	○	○	○	○	○

X→

Evidence-based testing is **necessary** in order for workplace genetic testing to be offered.

○ No, I disagree.

○ Yes, I agree.

Please select "next page" to continue.

End of Block: Section II: EBT

Start of Block: Section II: Health care integration

X→

Health care integration: Measures to ensure that workplace genetic test results can be integrated into the healthcare system and/or the electronic medical record.

	Strongly disagree	Somewhat disagree	Neither agree nor disagree	Somewhat agree	Strongly agree
Health care integration is **important.**	○	○	○	○	○
Health care integration is **likely to be achieved.**	○	○	○	○	○

X→

Health care integration is **necessary** in order for workplace genetic testing to be offered.

○ No, I disagree.

○ Yes, I agree.

Please select "next page" to continue.

End of Block: Section II: Health care integration

Start of Block: Section II: User-friendliness

X→

User-friendliness: Measures to ensure the workplace genetic testing program has a simple and easy-to-use design.

	Strongly disagree	Somewhat disagree	Neither agree nor disagree	Somewhat agree	Strongly agree
User-friendliness is **important.**	○	○	○	○	○
User-friendliness is **likely to be achieved.**	○	○	○	○	○

User-friendliness is **necessary** in order for workplace genetic testing to be offered.

- No, I disagree.
- Yes, I agree.

Please select "next page" to continue.

End of Block: Section II: User-friendliness

Start of Block: Section II: Utility for employers

Utility for employers: Measures to ensure **employers** can reap benefits through offering workplace genetic testing to their employees

	Strongly disagree	Somewhat disagree	Neither agree nor disagree	Somewhat agree	Strongly agree
Utility for employers is **important.**	○	○	○	○	○
Utility for employers is **likely to be achieved.**	○	○	○	○	○

Utility for employers is **necessary** in order for workplace genetic testing to be offered.

- No, I disagree.
- Yes, I agree.

Please select "next page" to continue.

End of Block: Section II: Utility for employers

Start of Block: Section II: Utility for laboratories

X→

Utility for laboratories: Measures to ensure **laboratories** can reap the benefits of performing and/or offering services related to workplace genetic testing.

	Strongly disagree	Somewhat disagree	Neither agree nor disagree	Somewhat agree	Strongly agree
Utility for laboratories is **important.**	○	○	○	○	○
Utility for laboratories is **likely to be achieved.**	○	○	○	○	○

X→

Utility for laboratories is **necessary** in order for workplace genetic testing to be offered.

○ No, I disagree.

○ Yes, I agree.

Please select "next page" to continue.

End of Block: Section II: Utility for laboratories

Start of Block: Section III: Final Questions

X→ X→

Assuming the key characteristics you described as NECESSARY were met, please now rate your agreement with the following statements:

	Strongly disagree	Somewhat disagree	Neither agree nor disagree	Somewhat agree	Strongly agree
Employers **should offer** workplace genetic testing.	○	○	○	○	○
Employers **should not be allowed** to offer workplace genetic testing.	○	○	○	○	○
Employers **should have the option** to offer workplace genetic testing.	○	○	○	○	○

Please select "next page" to continue.

Start of Block: Thank you!

Thank you for participating in our study. You will receive your Amazon e-gift cards in the amount of $25 for completing this survey and $100 for completing all three steps of our study within the next month.

End of Block: Thank you!

MODIFIED DELPHI PROCESS METHODS

Participants and recruitment

List of potential stakeholders

The research team (E.C., W.G.F., A.E.R.P., K.R., K.S., A.V.) developed a list of potential stakeholders for participation in the study from important stakeholder groups through an iterative process: employees (full-time workers and/or labor organization representatives), employers (managers of companies and/or business organization representatives), healthcare professionals (including healthcare organization representatives and clinicians), genetic testing

industry or insurance professionals (health, life, or disability insurance), and policy experts or ELSI professionals (bioethics, legal, or privacy experts and/or policy makers). The research team compiled a list of potential stakeholders organized by stakeholder category, including individuals and key organizations with publicly available contact information. The broader R01 research team and advisory council representing the stakeholder categories provided additional potential stakeholders. Potential stakeholders for participation in the study were prioritized based on types of perspectives they might represent as well as demographic variables such as years of experience, education, race/ethnicity, gender to try to maximize perspectives represented within stakeholder groups.

Screening potential stakeholders for study participation

Personalized invitations were sent up to three times per potential stakeholder, per online platform (e.g., email, LinkedIn). The screener (programmed in Qualtrics) identified potential stakeholders' self-reported stakeholder category from among the curated list (e.g., employees, employers, ELSI professionals, genetic testing industry representatives, and healthcare professionals). The screener also collected self-reported gender, race and ethnicity, and US region through multiple-response (select-all-that-apply) questions and perspectives on the selected stakeholder category through free-text responses. Given the broad spectrum of potential stakeholders in the "employee" stakeholder category, an additional approach was utilized to screen employees. The University of Michigan Health Research website (umhealthresearch.org) was used to advertise the study, and included a link to the study screener. This secure, password-protected database has a pool of over 90,000 individuals who have consented to be contacted for research opportunities.

The study invitation and the screener facilitated snowball recruitment of additional experts by forwarding the invitation within their professional network and soliciting contact information for potential stakeholders respectively. Respondents to the screener needed to be fluent in English, aged 18 years or older, have access to a computer or tablet that allowed video

conferencing, have internet access, and belong to one or more of the stakeholder categories to be eligible to participate in this study.

Selecting study invitees and final study participants

The research team that included a primary care provider, legal scholar, qualitative researcher, and genetic counselors selected the final list of potential stakeholders to be invited to participate in the study. The study consent form which detailed the entire modified Delphi process including the immediate next step of administering Survey 1 was sent by email to the selected study invitees. Those who completed the consent form followed by Survey 1 were considered to be enrolled study participants.

Modified Delphi Procedure and Materials

Survey 1 (June 2023)

Each participant completed a 20-minute online initial mixed-methods survey about their individual perspectives regarding employers offering genetic testing. Survey 1 began with an informational video created by the genetic counselor on the research team (E.C.) to introduce participants to the concept of wGT. We ascertained primary stakeholder role, years of experience, highest level of education, and age. Participants were then asked to list and rank, through free-text responses, up to five (a) potential benefits of wGT, (b) potential harms of wGT, and (c) potential design features important to consider for wGT. Survey 1 also included two Likert-type scale questions to assess participants' level of agreement on a 5-point scale (*1=Strongly agree, 5=Strongly disagree*) with the following statements: "*Employers should offer workplace genetic testing*" and "*Employers should NOT be allowed to offer workplace genetic testing*."

Deliberative Workshop (September 2023)

The 5-hour online Deliberative Workshop included three plenary sessions and two break-out (small group) discussions.

<u>Plenary session 1: Expert presentations</u>.

In the first plenary session, W.G.F. and A.E.R.P. (experts) provided educational presentations on workplace wellness programs, wGT, and relevant laws such as the Genetic Information Nondiscrimination Act and the Americans with Disabilities Act. They also shared the aggregate, de-identified results of Survey 1 with participants, including the top ranked benefits, harms, and design features of wGT. Each presentation was followed by a brief discussion/question and answer period and provided context for the subsequent small group session. The experts were also available to answer questions during the small group discussions if requested by the small group.

Small group discussions. Participants were assigned to one of five small groups led by a trained facilitator. Small groups were broadly segregated by stakeholder groups (employee, employer, ELSI scholar, genetic testing industry representative, healthcare professional). Prior to the workshop, facilitators attended a 2-hour training session with the research team that provided an overview of the research topic, the modified Delphi process, the Deliberative Workshop agenda, and a review of the small group discussions that they would be facilitating. The first small group discussion focused on the benefits and harms of wGT. Participants were shown Survey 1 qualitative data on the proposed or ranked benefits and harms of wGT to deliberate in small groups. Similarly, the second small group discussion focused on design features for wGT.

Plenary Sessions 2 and 3. In the second and third plenary sessions, each trained facilitator provided a summary of the content of their assigned small group's discussion. During the second plenary session, participants were given an opportunity to ask additional questions and provide responses to the report-backs. During the third plenary session, participants were shown data in aggregate from Survey 1 regarding the Likert-type scale questions. They were then asked to provide further commentary on their views and how their perspectives may have changed since the time of Survey 1. A.E.R.P. and W.G.F. moderated the discussion for plenary sessions 2 and 3.

Post-workshop evaluation. A short post-workshop evaluation was administered to determine if group dynamics could have impacted the results of the workshop.

Survey 2 (April 2024)

Survey 2 included the introductory YouTube video (shown in Survey 1). Participants were then asked to view the aggregate data from Survey 1 and re-rate their agreement with statements about employers offering/being allowed to offer wGT based on their current opinion. Participants were then asked to rate their agreement with five statements on secondary use of wGT results. Next, participants were asked to rate their agreement on statements on wGT about the importance and likelihood of being achieved. All agreement ratings were based on a 5-point scale that ranged from 1=*Strongly agree* to 5=*Strongly disagree*. For assessing participants' agreement on necessity (in order for wGT to be offered) for each of twelve "key characteristics" that could potentially maximize the benefits and minimize the harms of wGT, we deployed a binary response option (*1=Yes, I agree, 2=No, I disagree*). The 12 key characteristics for assessment were informed by findings from the deliberative workshop. Finally, participants were asked to again answer the questions about employers offering/being allowed to offer wGT, assuming the key characteristics they described as "necessary" in order for wGT to be offered were met.

Data Analysis

Survey Analysis

For Survey 1, each research team member reviewed up to five initial survey responses and made note of themes for the purpose of codebook development. Responses pertaining to benefits, risks and design features of wGT were included in the initial review and themes relevant to the key research questions were included in the codebook. The codebook was then comprehensively reviewed by the entire research team multiple times to ensure appropriate focus, accuracy. Benefits, risks, and design features of wGT were thematically coded in real-time by a qualitative researcher (K.R.) and a genetic counselor (E.C.) during an online team

meeting using MAXQDA software (VERBI Software, 2021). A lawyer with expertise in health law and ELSI (A.E.R.P.) then provided a final review of responses that were unclear or fell into an uncaptured theme. Furthermore, a single coder (E.C.) applied rank codes (1-5) based on survey responses, and overlap among the thematic codes and the rank codes was reviewed by the research team to determine which themes were most frequently ranked 1-5 for benefits, risks and design features. Data generated from this analysis was shown to participants during the deliberative workshop.

The rest of the quantitative survey data from Survey 1 and Survey 2 of the modified Delphi process were summarized using descriptive statistics. A biostatistician (S.P.) applied Chi-square tests to analyze differences between stakeholder groups with respect to (a) agreement with the necessity of each of the 12 key characteristics, and (b) distribution of agreement items about employers offering/being allowed to offer wGT from the first portion of Survey 2.

We undertook several types of analyses to track participant responses to the questions about whether employers should offer/be allowed to offer wGT. Descriptively, we created five categories of responses based on the Likert-type scale scores for the two normative questions: 1) supportive, 2) permissive, 3) neutral/conflicted, 4) libertarian, and 5) opposed. These categories were finalized by comparing both quantitative Likert-type scale scores to qualitative open-ended responses. We then assessed the extent to which participants changed their opinions across Survey 1, the beginning of Survey 2, and the end of Survey 2 (when the design features for wGT were assumed). Furthermore, Chi-square analysis was used to determine if there were significant differences between participants' likelihood of moving towards agreement or disagreement with questions about employers offering/being allowed to offer wGT from (a) Survey 1 (pre-workshop) to the beginning of Survey 2 (post-workshop), (b) Survey 1 (pre-workshop) to the end of Survey 2 (post-workshop, assuming key characteristics met), and c) the

beginning of Survey 2 (post-workshop) to the end of Survey 2 (post-workshop, assuming key characteristics met).

Deliberative Workshop Analysis

Audio recordings of the small group discussions and plenary sessions were transcribed verbatim and de-identified by LandMark Associates, a HIPAA-compliant transcribing service. Each research team member (A.E.R.P., W.G.F., K.R., K.S., E.C.) reviewed 2-4 transcripts each, and created a memo for each transcript. Memos were reviewed with attention to recommendations for wGT design features and key characteristics for implementation of wGT. From the memos, twelve “key characteristics” for maximizing the benefits and minimizing the potential harms of wGT were identified. Representative quotes were collected by K.R. for each key characteristic.

SUPPLEMENTAL TABLES AND FIGURES

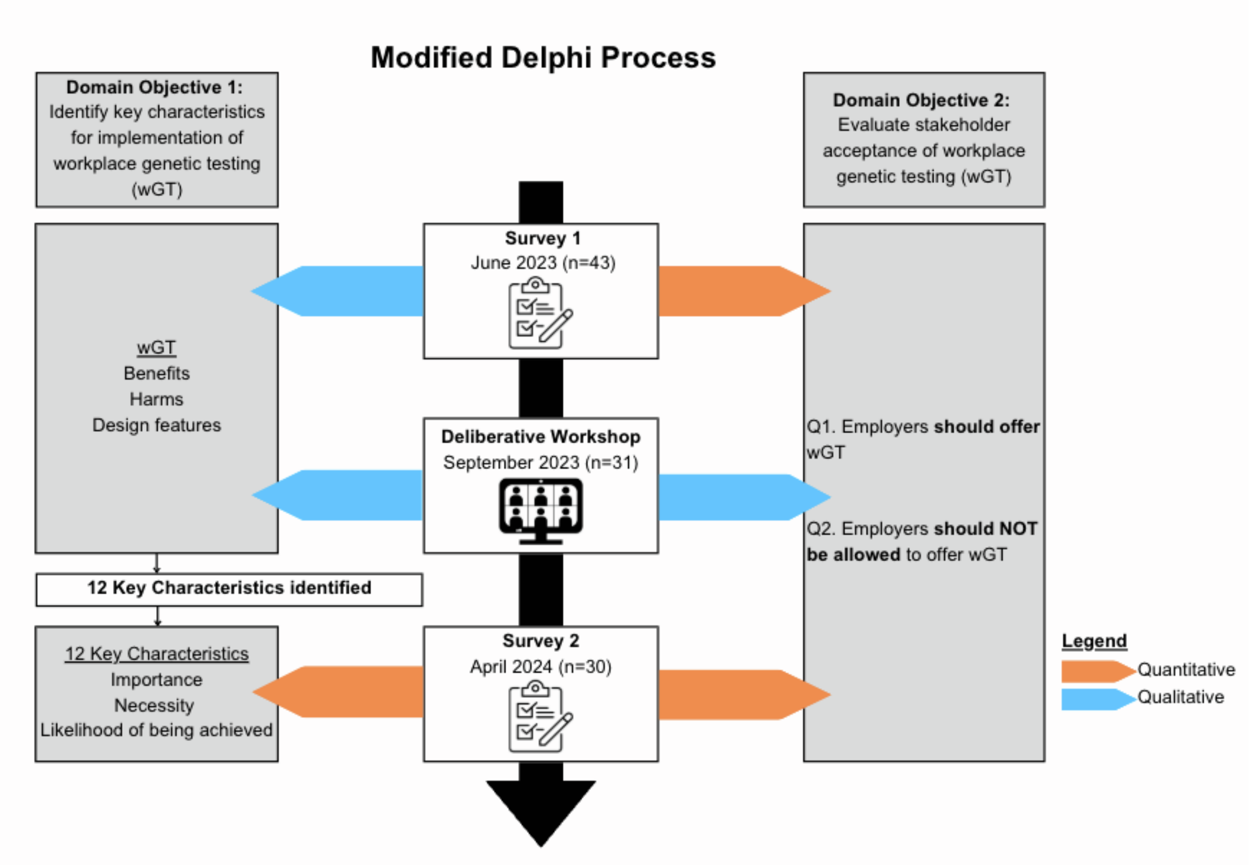

Supplemental Figure 1. Overview of the modified Delphi process. Important stakeholder perspectives on (1) key characteristics for implementation of workplace genetic testing (wGT) and (2) acceptance of wGT were evaluated through a three-round, mixed-methods Delphi process including Survey 1, a Deliberative Workshop, and Survey 2 (See Methods and additional details about methods described in Supplemental Materials).

Supplemental Table 1. Summary of research participants responding through the modified Delphi process.

Stakeholder Category	N (%)<br>Survey 1<br>(N=43)	N (%)<br>Deliberative Workshop<br>(N=31)	N (%)<br>Survey 2<br>(N=30)
Employee	8 (19%)	6 (19%)	6 (20%)
Employer	8 (19%)	4 (13%)	4 (13%)
ELSI professional	10 (23%)	9 (29%)	8 (27%)
Genetic testing company/ industry representative	8 (19%)	8 (26%)	8 (27%)
Healthcare professional	9 (21%)	4 (13%)	4 (13%)

Supplemental Table 2. Benefits and risks for workplace genetic testing (wGT) emerging from participants' free-text responses from Survey 1 (codebook for Survey 1) (n=43).

Benefits	Definition
Top benefits	
Access	Any mention of improved access to genetic services or testing, including through the reduction of barriers, increased convenience; Genetic testing leading to access to support or resources; Making it easier to receive genetic testing; convenient access
Health impacts	Any mention of changing employee healthcare behaviors, informing employees that they need to engage in healthcare behaviors (such as screening, testing, medication change, etc.), or engagement with healthcare providers; proactive in healthcare system OR any mention of positive health impact, health improvement or improved clinical outcome for employee; personalized or targeted therapy; Preventing negative health outcomes; Early detection and intervention
Knowledge	Any mention of employee having a greater awareness or knowledge of genetics, genetic risk, "knowledge is power" sentiments; Increasing knowledge of genetics in general; Awareness or curiosity
Some mentioned	
Family health impacts	Any mention of positive health impact for the participant's family, including cascade testing or screening, prevention, planning, etc.; Family receiving information about health and risks; reproductive implications of genetic testing
Positive emotions	Any mention of positive employee reactions, such as peace of mind, reduced anxiety, increased satisfaction, feeling motivated, relief, etc.
(Reduced) cost	Any mention of being free or low cost to employee; discounted cost; reduced insurance premiums, and incentives that reduce cost
Few discussed	
De-stigmatization	Any mention of normalizing or destigmatizing genetic testing or participating in genetic testing
Financial planning	Any mention of being proactive/planning for the future (i.e., finances, disability, insurance) and explicit reference to expenses related to health
Lifestyle/behavior impacts	Any mention of changing employee lifestyle behaviors, or informing employee that they need to engage in lifestyle behaviors (such as diet, exercise, reducing stress, etc.); proactive in lifestyle changed
Employee recruitment and retention	Any mention of increasing employee satisfaction or loyalty; demonstrating that the employer cares about employees; retaining employees OR any mention of wGT being a differentiator or an enticing option for potential employees, improving recruitment or recruitment advantage
Reducing employer costs	Any mention of reducing costs; reducing insurance costs to employer; reducing absenteeism/sick days; healthier employees leading reduced costs
Business planning	Any mention of a benefit to the employer for business planning purposes, including benefits planning, or preparing for future business or benefits needs
**Risks**	**Definition**
Top risks	
Discrimination	Any mention of employees experiencing insurance discrimination (health, disability, life insurance), increased insurance cost or denial of coverage OR any mention of employees experiencing employment discrimination; Not receiving promotion; Not being hired; being treated unfairly by employers

Lack of access to follow-up	Any mention of employees either lacking access to follow-up, being unable to afford follow-up, not knowing what to do for follow-up healthcare or genetic counseling, or receiving incorrect or costly follow-up care; Lack of guidance regarding follow-up care; Lack of or inadequate post-test genetic counseling
Lack of understanding	Any mention of employees receiving inadequate education or lack of understanding of genetics, genetic testing, or genetic results, or misunderstandings; False sense of security; Genetic determinism; Results are too complicated to understand; inadequate or lack of pretest education/counseling
Negative emotions	Any mention of negative employee reactions, such as anxiety, depression, hopelessness, feeling stigmatized, traumatized by results, uncertainty, etc.
Privacy-related risks	Any mention of employee concerns about privacy, confidentiality, and invasions of privacy. Who has access to the data by design?
Some mentioned	
Coercion	Any mention of a employees feeling coerced or pressured into genetic testing by the employer
Health mismanagement	Any reference to unnecessary or harmful medical screenings, surveillance, surgeries, etc. based on results; Inappropriate lifestyle or health decision-making by patient or provider
Inaccurate testing	Any mention of employees receiving or misleading or inaccurate results; False positive or false negatives
Security-related risks	Any mention of hacking, leaked data, encryption, protocols for ensuring security of data; stopping unintended access
Few discussed	
Inconclusive results/VUS	Any mention of VUS (variants of uncertain significance) or inconclusive genetic test results
Lack of test actionability	Any discussion of lack of evidence for testing or no ability to act on results
Risks to employees' family members	Any discussion of family in the risks section, including risk to family
Risks to the employer	Any mention of negative impact to the employers, including sunk investment in the program, cost, or low participation, liability, lower employee satisfaction or trust
Secondary use of data	Any mention of secondary use by a third party or selling of employee data; Lack of control by employee of what happens to their data; misuse of data by employers if discrimination was not specifically mentioned
Stigma	Any mention of stigma, loss of reputation, or being looked down upon for genetic testing, participating in genetic testing, or genetic testing results

Supplemental Table 3. Results of post-workshop evaluation (n=29).

Statement	N (%)				
	Strongly Agree	Somewhat Agree	Neither Agree nor Disagree	Somewhat Disagree	Strongly Disagree
Overall, I was satisfied with the interactive workshop.	16 (55%)	10 (34%)	--	2 (7%)	1 (3%)
My viewpoint was taken seriously during the workshop.	25 (86%)	2 (7%)	--	1 (3%)	1 (3%)
My opinions were respected by my small group.	26 (90%)	2 (7%)	--	1 (3%)	--
My perspectives and participation made an impact on the interactive workshop.	15 (52%)	10 (34%)	2 (7%)	1 (3%)	1 (3%)
There were diverse stakeholder perspectives at the workshop.	17 (59%)	8 (28%)	2 (7%)	1 (3%)	1 (3%)
My time participating in this workshop was worthwhile.	15 (52%)	11 (38%)	3 (10%)	--	--
I felt comfortable participating in the interactive workshop.	23 (79%)	4 (14%)	2 (7%)	--	--
The presenters were unbiased on the topic.	26 (90%)	1 (3%)	1 (3%)	1 (3%)	--
I would abide by the group's final decision, even if it differs from my personal opinion.	6 (21%)	5 (17%)	10 (34%)	5 (17%)	3 (10%)
I felt that my group had a good discussion, even if I personally held a different viewpoint than my other small group members.	25 (86%)	3 (10%)	1 (3%)	--	--
Differences in power between the stakeholders negatively affected the conversation in my small group.	--	--	4 (14%)	4 (14%)	21 (72%)

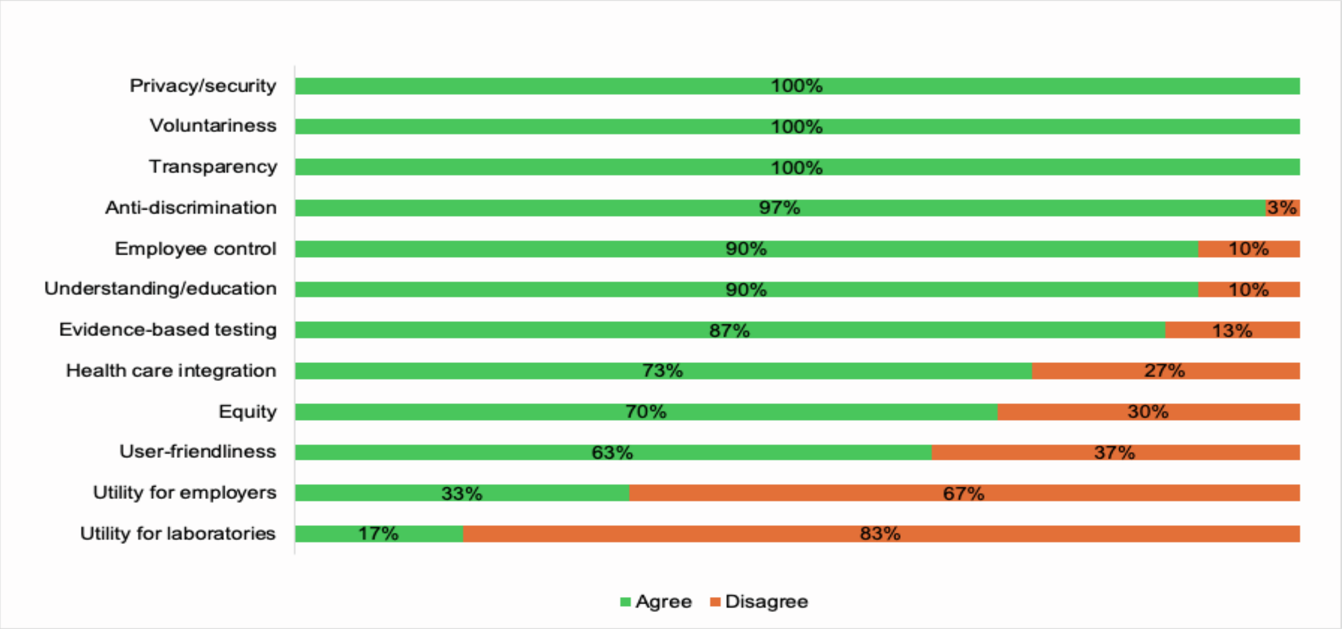

Supplemental Figure 2. Participant perspectives on the <u>necessity</u> of key characteristics in order for wGT to be offered (n=30).

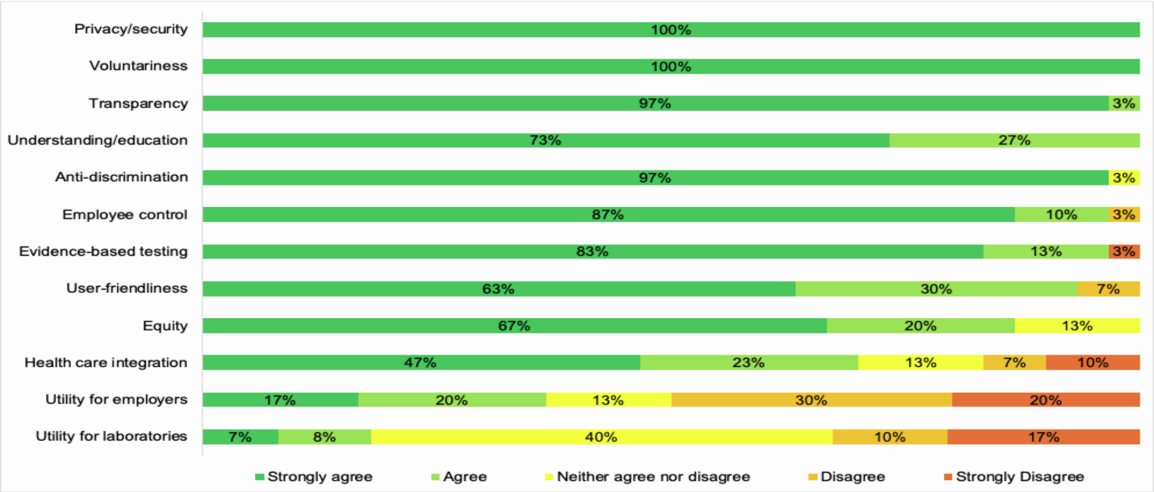

Supplemental Figure 3. Participant perspectives on the importance of key characteristics for wGT (n=30).

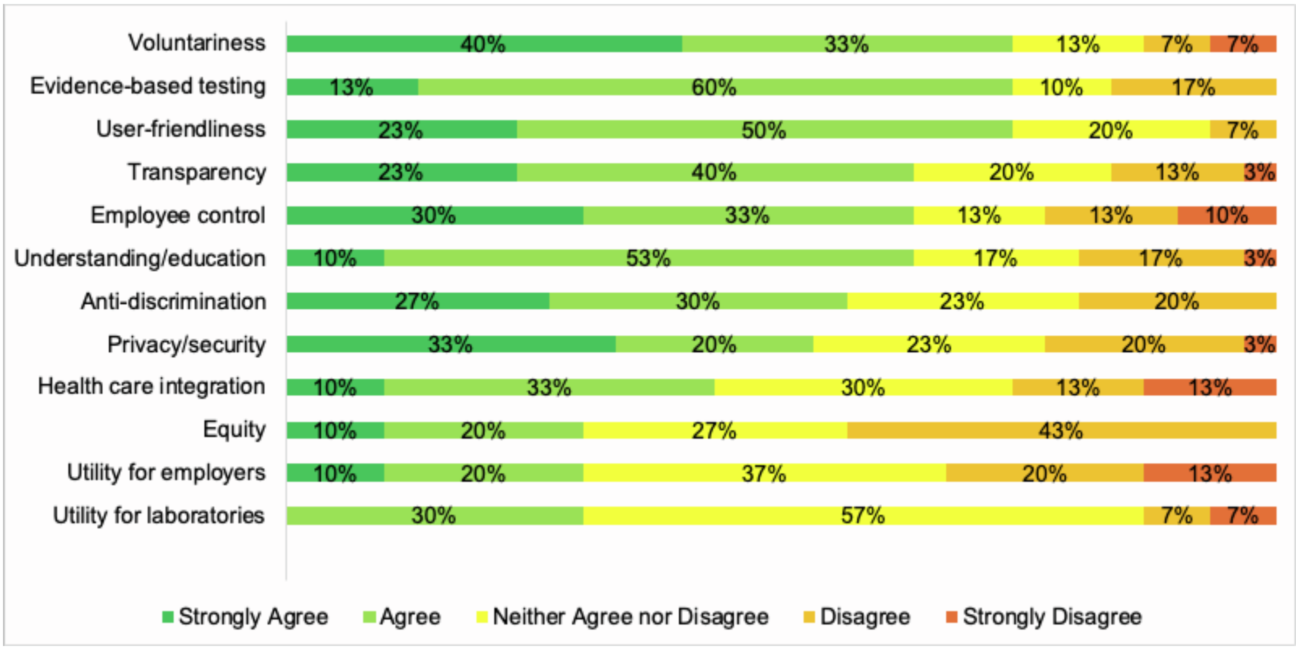

Supplemental Figure 4. Participant perspectives on the likelihood of key characteristics for wGT being achieved (n=30).
