# Supplementary material for: Multidisciplinary stakeholder-informed identification of key characteristics for implementation of workplace genetic testing
Source: HGG Adv. 2025 May 22;6(3):100458. doi: 10.1016/j.xhgg.2025.100458 (PMC12169765; doi:10.1016/j.xhgg.2025.100458)
Supplement: Document S2. Article plus supplemental information [file mmc2.pdf]

HGG Advances
ARTICLE

Multidisciplinary stakeholder-informed identification of key characteristics for implementation of workplace genetic testing

Elizabeth Charnysh,[1] Kunal Sanghavi,[1,9,*] Kerry A. Ryan,[2] Alyx Vogle,[3] Alexandra Truhlar,[1] Subhamoy Pal,[4] Jonathan M. Reader,[4] J. Scott Roberts,[5] Charles Lee,[1] Anya E.R. Prince,[6,8] W. Gregory Feero,[7,8] and INSIGHT @ Work Consortium

Summary

Workplace genetic testing (wGT) is an evolving model for genetic testing where employees are offered consumer genetic testing through employer-sponsored wellness programs. However, the potential harms, benefits, and key characteristics for best implementation practices for wGT have yet to be defined. To address this issue, we conducted a three-round modified Delphi process, including multiple rounds of survey and a virtual deliberative workshop, with purposely chosen wGT stakeholders (employees, employers, ethical, legal, and social implications [ELSI] professionals, genetic testing industry representatives, and healthcare professionals) to share their perspectives. From the modified Delphi process, we identified 12 key characteristics for the implementation of wGT that were perceived to increase the potential for benefit while reducing the risk of potential harms. Most participants agreed that privacy/security, voluntariness, transparency, understanding and education, anti-discrimination, employee control, and evidence-based testing measures were both important (>90%) and necessary (>75%) for the implementation of wGT. However, some participants also expressed a lack of confidence in the likelihood of achieving these characteristics in wGT programs. Overall, stakeholders expressed qualified support for wGT at the conclusion of the modified Delphi process. Their perspectives on the topic varied over the course of the process and were at least partially contingent on whether the aforementioned 12 key characteristics were met. These findings help inform the establishment of a normative framework for wGT assessment.

Introduction

Clinical, public, and private predictive genetic testing service providers have long had the goal of increasing access to genetic testing given its potential to inform disease prevention and screening.[1–3] Avenues for genetic testing have shifted dramatically over the past few decades, from testing primarily taking place in clinical settings,[4] to now having consumer genetic testing options outside healthcare systems, such as direct-to-consumer genetic testing (dtcGT), where consumers order testing directly without involvement of a clinician,[4] and consumer-initiated genetic testing (ciGT), where the laboratory assigns a clinician to be the ordering provider for a consumer's test order.[5] More recently, the workplace has emerged as another access point for consumer genetic testing, where genetic testing is offered through employer-sponsored wellness programs, otherwise known as workplace genetic testing (wGT).[6–10] Some major companies have shared publicly that they have implemented wGT.[6,11–14] While one study identified lack of evidence of broad uptake of wGT in the United States,[11] it has been challenging for researchers to fully analyze the evolving landscape.[11] Typically, wGT is offered at no cost to employees, is performed by a consumer genetic testing laboratory, and includes multi-gene panel testing for a range of conditions and purposes (e.g., hereditary cancer syndromes, hereditary cardiovascular disease, and medication response [pharmacogenomics]).[9] One study examining employees of a large US healthcare system reported that approximately half of survey respondents indicated they pursued wGT and received their test results, and that those who received results indicating they were at increased risk for cancer or heart disease were more likely to change their health behaviors or utilize healthcare services.[9] However, other studies published to date have mostly explored employees' perspectives on a hypothetical wGT scenario.[7,15,16]

As genetic testing moves into the workplace, it is important to examine the potential benefits and harms in this context. While there have been limited studies on wGT specifically, previous research on consumer genetic testing can offer insights into the potential benefits and harms of wGT. Consumer genetic testing has been shown

[1]The Jackson Laboratory for Genomic Medicine, Farmington, CT, USA; [2]Center for Bioethics and Social Sciences in Medicine, University of Michigan School of Medicine, Ann Arbor, MI, USA; [3]Department of Cardiology, Brigham and Women's Hospital, Boston, MA, USA; [4]Michigan Alzheimer's Disease Research Center, Department of Neurology, University of Michigan School of Medicine, Ann Arbor, MI, USA; [5]Department of Health Behavior and Health Equity, University of Michigan School of Public Health, Ann Arbor, MI, USA; [6]University of Iowa College of Law, Iowa City, IA, USA; [7]Maine Dartmouth Family Medicine Residency, Augusta, ME, USA
[8]Senior author
[9]Lead contact
*Correspondence: kunal.sanghavi@jax.org
https://doi.org/10.1016/j.xhgg.2025.100458.
© 2025 The Author(s). Published by Elsevier Inc. on behalf of American Society of Human Genetics.
This is an open access article under the CC BY license (http://creativecommons.org/licenses/by/4.0/).

to increase awareness and access to genetic testing for those who may not otherwise meet clinical criteria or have ready access to clinical genetics services,[17–19] as well as increase the overall ease of use of genetic testing.[3] However, consumer genetic testing is only available to those who have the means to pay for it, raising concerns about the potential for worsening socioeconomic disparities.[20–23] A myriad of other challenges with consumer genetic testing have been identified, including the lack of genetic counseling typically included in consumer genetic testing workflows,[24,25] false reassurance and confusion about risk information,[3,26,27] the mixing of medical findings with ancestry and non-medical information,[28] integration of results into the medical record and appropriate follow-up care,[29] and privacy and secondary data sharing/use concerns.[30–32]

In general, workplace wellness programs have their own set of ethical, legal, and social implications (ELSI). Often touted as a "win-win"[33] for the employer and employee given their potential to expand access to actionable health information, these programs have raised questions and concerns about effectiveness,[34–37] privacy and data sharing,[38–40] and whether incentives are truly rewards ("carrots"), or actually discriminatory penalties ("sticks").[33,40,41] Introducing genetic testing as a wellness benefit adds additional complexity to these programs given the sensitivity and nature of genetic information as well as the nuanced policies and laws surrounding it. For example, while the Genetic Information Nondiscrimination Act (GINA) generally prohibits employers from collecting genetic information from their employees and discriminating against them based on genetic information, GINA has an exception allowing employers to collect employee genetic information in a deidentified, aggregate manner as part of workplace wellness programs.[6]

Currently, there are no policy guidelines for the assessment and implementation of wGT.[6] One recent study reported on programmatic design features that could reduce barriers to employee participation in a hypothetical wGT program based on a survey of employed US adults.[16] Briscoe and colleagues identified the ability to later delete one's data from the wGT program, policies prohibiting data sale or sharing, and control over how employee data are used to be design features most likely to increase respondents' likelihood of participating in a theoretical wGT program.[16] Existing frameworks for ethical implementation of consumer genetic testing have prioritized informed consent, respect for privacy and confidentiality, genetic counseling, and ensuring clinical utility and validity, among other criteria.[42–44] However, understanding which key characteristics are important to the ethical design and implementation of a wGT program from the perspectives of stakeholders is critical. Key characteristics, as identified by stakeholders directly involved with or affected by wGT, can inform policy deliberations, normative assessment, and wGT program implementation. To this end, we conducted a modified Delphi process with multiple survey rounds and a deliberative workshop to assess acceptance of wGT from the perspectives of important stakeholders and to identify key characteristics that have the potential to enhance benefit of wGT to individuals and society.

Methods

Participants, recruitment, and study design

Important stakeholder groups were identified by the research team (E.C., W.G.F., A.E.R.P., K.A.R., K.S., and A.V.) and through review of relevant literature.[6] Stakeholder groups included (1) employees (full-time workers and/or labor organization representatives), (2) employers (managers of companies and/or business representatives), (3) ELSI professionals (bioethics, legal, privacy, or policy experts), (4) genetic testing industry representatives (commercial genetic testing laboratory representatives, commercial genetic counseling service providers, and health insurance industry professionals), and (5) healthcare professionals (genetic counselors, physicians, nurses, other clinicians). Consistent with similar studies involving Delphi methodology,[45–51] we aimed to recruit 40–50 research participants for the study. Potential individuals representing each stakeholder group were initially identified by the research team with the input of an expert scientific advisory board composed of ELSI experts, followed by snowball sampling. Participants were then prioritized for enrollment based on personal characteristics such as years of experience, education, race and ethnicity, and gender collected through the study screener to try to maximize the diversity of perspectives represented within stakeholder groups. Participants provided written consent before participation. Participants received a total of $300 for their full participation.

A modified Delphi process was carried out from June 2023 to April 2024 (Figure S1) and included (1) an initial 20-min online survey (Survey 1, Summer 2023); (2) a 5-h online interactive Deliberative Workshop (Fall 2023); and (3) a 20-min online follow-up survey (Survey 2, Spring 2024). Delphi methods were modified and adapted from those of other studies with a similar design[45–47] and specific methodology guidance.[48–51] The study was organized around two primary objectives pertaining to research domains: (1) the identification of key characteristics for implementing wGT, and (2) an evaluation of stakeholder acceptance of wGT. This study was determined to be exempt from institutional review board (IRB) oversight by the IRBs of The Jackson Laboratory, The University of Michigan, The University of Iowa, and MaineGeneral Medical Center. Additional details about the methods used for the modified Delphi process and an overview of the process (Figure S1) can be found in the supplemental materials. Participants were required to have completed each prior step in order to continue participating in the study.

The research team sent eligibility screeners to 139 potential stakeholders, including employees, employers, ELSI professionals, healthcare professionals, and genetic testing industry representatives. This approach yielded $n = 96$ screened eligible potential stakeholders for research participation. From this list, the study team selected $n = 53$ potential stakeholders to send the study consent and Survey 1, prioritizing diversity in types of stakeholder perspectives and demographic characteristics.

Table 1. Categories of stakeholder acceptance of workplace genetic testing (wGT) and associated response criteria

Category	Response criteria[a] Q1. Employers should offer wGT	Q2. Employers should NOT be allowed to offer wGT
Supportive	somewhat agree or strongly agree	somewhat disagree or strongly disagree
Permissive	neither agree nor disagree	somewhat disagree or strongly disagree
Neutral	neither agree nor disagree, somewhat disagree, or strongly disagree	neither agree nor disagree
Libertarian	somewhat disagree or strongly disagree	somewhat disagree or strongly disagree
Opposed	somewhat disagree or strongly disagree	somewhat agree or strongly agree

[a]Likert-scale answer choices for Q1 and Q2 included the following: Strongly agree, somewhat agree, neither agree nor disagree, somewhat disagree, strongly disagree.

The goal was to recruit approximately 8–10 participants in each stakeholder group, keeping the groups comparable in size.

Identifying key characteristics for implementation of wGT

Many of the items in Survey 1 were designed to elicit participants' initial perspectives on the potential benefits, harms, and programmatic design of wGT to provide the groundwork for future group discussions about potential key characteristics for wGT during the Deliberative Workshop. Survey 1 included free-text response items in which participants were asked to independently list and rank up to five (1) potential benefits of wGT, (2) potential harms of wGT, and (3) potential design features important to consider for wGT. The research team developed a codebook inductively based on participants' responses to Survey 1 as well as the principles of thematic analysis. Benefits, harms, and design features of wGT were thematically coded. The aggregated responses were analyzed to identify the most frequent themes among participants' responses.

During the Deliberative Workshop, two research team members (W.G.F. and A.E.R.P.) provided educational presentations on workplace wellness programs, wGT, and relevant laws (e.g., GINA) and shared the aggregate, deidentified results of Survey 1 with participants, including the top ranked benefits, harms, and design features important to consider for wGT. Participants were assigned to one of five small groups, broadly segregated by stakeholder groups (employee, employer, ELSI scholar, healthcare provider, genetic testing industry representative). Small group discussions focused on benefits, harms, and design features for wGT. Given the inclusion of stakeholders with diverse experiences and expertise, brief post-workshop evaluation was developed based on content from other studies.[46,47,52] The post-workshop evaluation was administered immediately after the Deliberative Workshop to assess stakeholder satisfaction and comfort level during the workshop, perceptions of bias from the research team, and group dynamics. Audio recordings of the Deliberative Workshop were transcribed verbatim and deidentified by Landmark Associates. Research team members created short qualitative summaries for each transcript. These summaries were reviewed with attention to recommendations for the design and implementation of wGT to maximize benefits and minimize harms. From this information, 12 key characteristics were identified by the research team and representative quotes were collected for each key characteristic by a qualitative researcher (K.A.R.).

In Survey 2, participants were presented with each of the 12 key characteristics in a random order and asked to rate their agreement with the (1) importance, (2) likelihood of being achieved (Likert scale: 1 = *Strongly agree*, 5 = *Strongly disagree* for importance and likelihood of being achieved), and (3) necessity in order for wGT to be offered (1 = *Yes, I agree*, 2 = *No, I disagree*) for each characteristic. These data were summarized with descriptive statistics.

Evaluating stakeholder acceptance of wGT

We evaluated stakeholder perspectives on acceptance of wGT (e.g., whether it should be offered by employers, or allowed to exist altogether), and how their perspectives changed over the course of the modified Delphi process. Likert-scale questions were administered at multiple stages of the process: During Survey 1 and at the beginning of Survey 2, participants were asked to rate their agreement on a 5-point Likert-type scale (1 = *Strongly agree*, 5 = *Strongly disagree*) with two statements deliberately designed to be dichotomizing regarding acceptance of wGT: (Q1) *Employers should NOT be allowed to offer wGT*; (Q2) *Employers should offer wGT*. During the Deliberative Workshop, one plenary session was dedicated to discussion on these questions about acceptance of wGT. Data on Q1 and Q2 were presented in an aggregate, deidentified form to participants during the Deliberative Workshop and in Survey 2. At the end of Survey 2, participants were asked to respond to these two questions again, this time, assuming hypothetically that the key characteristics they previously indicated were "necessary for wGT to be offered" had been met.

We undertook several types of analyses to analyze participant responses to the items assessing stakeholder acceptance of wGT over the course of the modified Delphi process. Only those participants who completed all three rounds of the process were included in these analyses. First, we inductively created five descriptive categories of responses to these items: (1) Supportive (disagreed on the Likert scale that employers *should not be allowed to offer* and agreed that employers *should offer* wGT), (2) permissive (disagreed that employers *should not be allowed to offer* and neutral that employers *should offer* wGT), (3) neutral/conflicted (neutral on whether employers *should be allowed to offer* wGT), (4) libertarian (disagreed that employers *should not be allowed to offer*, and disagreed that they *should offer* wGT), and (5) opposed (agreed that employers *should not be allowed to offer* and disagreed that they *should offer* wGT) (Table 1). The descriptive categories pertaining to participants' acceptance of wGT were reviewed for both participants who dropped out of the study after Survey 1 and those who completed the entire study.

Categories were assigned to participants' responses by comparing quantitative Likert-type scale scores to qualitative open-ended responses, highlighted by representative quotes.

Table 2. Survey 1 participant characteristics ($N = 43$)

Participant variable	*n* (%)
**Primary stakeholder group**[a]	
Employee	8 (19)
Employer	8 (19)
ELSI professional	10 (23)
Genetic testing company/industry representative	8 (19)
Healthcare professional	9 (21)
**Gender**	
Female	27 (63)
Male	15 (35)
Non-Binary	1 (2)
**Race and ethnicity**	
Asian	2 (5)
Black, non-Hispanic	2 (5)
Hispanic/Latino	5 (12)
White, non-Hispanic	31 (72)
More than one race and ethnicity	3 (7)
**Age, y**	
25–35	9 (21)
36–45	6 (14)
46–55	13 (30)
56–65	7 (16)
≥66	8 (19)
**Education**	
Postgraduate or professional degree	36 (84)
Four-year college or university degree	6 (14)
Some graduate school	1 (2)

[a]Multiple participants identified with more than one stakeholder group. The research team categorized employees into stakeholder groups based on publicly available information about each individual, which sometimes did not match the self-reported stakeholder group (e.g., some healthcare professionals selected "employee," but their professional healthcare experience was prioritized for this study).

Next, we assessed the extent to which participants changed their opinions throughout the modified Delphi process. Chi-square analysis was used to determine if there were significant differences in participants' likelihood of moving toward agreement or disagreement with these two statements at each step of the modified Delphi process.

Results

Study participants

A total of 43 participants consented and completed Survey 1 (Tables 2 and S1). The majority were White ($n = 31$ of 43, 72%), female ($n = 27$ of 43, 63%), and had a postgraduate or professional degree ($n = 36$ of 43, 84%) (Table 2). Thirty-one participants attended the Deliberative Workshop, and 30 participants completed Survey 2. The highest attrition was observed in the healthcare professional and employer stakeholder groups between Survey 1 and the Deliberative Workshop (Table S1). Anecdotally, the reasons most often given for dropping out in participants' communications to study investigators related to conflicting time commitments. Overall, the percentage of participants who began the study and indicated they were supportive or permissive of wGT on Survey 1, but did not complete the entire study, mirrored that of those who completed the entire study. Results of the post-workshop evaluation indicated that most participants either somewhat or strongly agreed that (1) they were satisfied with their experience ($n = 26$ of 29, 90%), (2) their viewpoint was taken seriously ($n = 27$ of 29, 93%), (3) they felt comfortable participating ($n = 27$ of 29, 93%), and (4) the research team presenters were unbiased on the topic ($n = 27$of 29, 93%) (Table S3).

Identifying key characteristics for implementation of wGT

During the Deliberative Workshop, participants were shown, in aggregate, their self-identified perceived benefits and harms of wGT from Survey 1. Top potential benefits of wGT raised by participants in Survey 1 included positive health outcomes, knowledge about genetic risk, and access to genetic testing. Top potential harms included privacy-related harms, discrimination, lack of understanding, negative emotions, and lack of access to follow-up care (full descriptions can be found in Table S2). During the Deliberative Workshop discussions of these findings, 12 key characteristics of benefit-maximizing and harm-reducing wGT programs emerged (Table 3). Small groups tended to focus on slightly different aspects or details of wGT in their Deliberative Workshop discussions; however, all of the groups endorsed and discussed in detail both the potential harms and potential benefits of wGT. We did not observe any overt differences among small group discussions in relationship to the identification of key characteristics for implementing wGT, or their overall assessment of wGT. Furthermore, when small group discussions were brought back and reported out to the larger group, additional deliberation tended to reveal commonalities, as opposed to divergences, in discussions.

While most participants agreed that privacy/security, voluntariness, transparency, understanding/education, anti-discrimination, employee control, and evidence-based testing were important (>90%) and necessary (>75%) for implementation of wGT, some expressed doubts about their likelihood of being achieved (Figures 1 and S4). Equity and healthcare integration were considered relatively important but were also seen as less likely to be achieved than the other key characteristics. In addition, utility for laboratories and utility for employers received low mean scores on both importance and likelihood of being achieved (Figure 1). Figures S2–S4 provide

Table 3. Twelve key characteristics identified through the modified Delphi process to maximize the potential benefits of workplace genetic testing (wGT) while minimizing potential harms

Key characteristic	Description: Measures to ensure …	Representative quote(s)
Anti-discrimination	… prevention of unfair treatment based on data from wGT.	ELSI stakeholder: *I would think that one of the biggest risks is what happens to that information and then how can it be used—essentially, how can it be used against me? Then not only me, but actually my family, and how does it impact my insurance rates? How does it impact my employability? Recognizing that there are laws that prevent discrimination, we also acknowledge that there are ways and loopholes that happen with laws all the time. For me, there would be a lot of fear or concern about having this information available to my employer … It's the fear of unknown and not knowing the details of the law and how to ensure that my employer sticks to it without having to get lawyers and get involved … It's a fear of, what do you do when something goes awry … it's a lot to have to take on and to monitor.*
Employee control	… employees are in charge of the management and use of data from wGT.	Employer stakeholder: *It is about the employee data control slash EMR integration … making sure that [the] employee has some way to get their data. They own it. They control it. Ideally, they can have access to the app in perpetuity.*<br>ELSI stakeholder: *I was just wondering if there would be the options of ability in how you would transfer information potentially to transition that into the clinical space 'cause a lot of—if it comes in as a—I know, when it comes to clinical testing, we have just PDFs, not very useful over the long term, but there are health systems that have more sustained decision support that's useful over the long term.*
Equity	… there is equitable access to wGT, related benefits, and follow-up.	Genetic testing industry stakeholder: *Going back to the disparities in terms of healthcare delivery. I think one big risk is that they get a result and then there's no bridge to any sort of care and particularly when—especially with bigger companies, there's wide variability in what health insurance plan somebody may have … if there's no confidence that if you come back with a BRCA1 mutation that you can go and get yourself a breast MRI for a reasonable price, or that you could get your ovaries removed, if you so choose … Those are things that will have the potential to cause a lot more harm for those individuals, once they try to get to the next step … A lot of people can fall off, and then that information no longer becomes useful.*<br>Employee stakeholder: *If you are offering a service where people can get genetically tested or whatever-not, are these employers, are they also going to offer, I don't know, a week's worth of, I don't know, or 3 days' worth of PTO around that so that maybe you can then make the follow-up visits to a doctor … ?*
Evidence-based testing	… testing is performed by a reputable laboratory and that what is analyzed on the test is supported by evidence and produces accurate results.	Genetic testing industry stakeholder: *I wanna see actionable evidence-based testing from a [Clinical Laboratory Improvement Amendments and College of American Pathologists (CLIA/CAP)-certified laboratory]. It falls under clinical laboratory testing rules and regulations.*<br>Employer stakeholder: *Yeah, put in a pitch for evidence-based, I think. That's because that's of big importance to me. Tests where you don't have a sufficient evidence base to understand the upsides and the downsides and how those might be balanced, I think, just makes for bad testing … There's a lot of information freely available in the literature and online about what … you might put together for a panel. You might have to delve in a little deeper to understand whether or not there's any … utility actually to using that panel over current clinical practice. Making sure that thought goes into what tests are included and how it's reported out. I think for me evidence-based is an essential item.*<br>Employee stakeholder: *I have one … accuracy. What is the accuracy of these tests? Are we talkin' 80%, 99%? What? Because if the results are not 100%, or close to it, accurate, then they're pretty worthless.*

(*Continued on next page*)

Table 3. ***Continued***

Key characteristic	Description: Measures to ensure …	Representative quote(s)
Healthcare integration	… that wGT results can be integrated into the healthcare system and/or the electronic medical record.	Genetic testing industry stakeholder: *Then the lack of access to follow-up I think is really a serious problem. There's no, if—if I should say—if there is no clear path to having had an employee wellness test, if something comes up that is of significance, there's a way to immediately get into counseling and follow-up care.*<br>Employee stakeholder: *Yeah, no, and there is that disconnect, right now, between this—these results and your normal health insurance because you may wanna have a follow-on test, but you need to then pass that along to your health insurance. I don't know. I don't think that happens through the employer 'cause they don't really—I mean, they're—the health insurance is a separate entity. It may be direct with the—I mean, I don't know. Maybe you have to sign a release of information to send it to your health insurance. I don't know, but I don't think it would be the employer.*
Privacy/security	… the confidentiality and security of wGT results.	Healthcare professional stakeholder: *… employers should not be able to get their hands on individualized data about their employees. That's actually in the laws. Still, that would be a prerequisite.*<br>Employee stakeholder: *I think we spoke a lot about, I think—what was it, breach of data? I think it was <other participant> who said that, and scared me, that it's hard to code things as private once you get that with a lot of data or participant information. I don't know. Going off of that, I would think privacy-related features, that makes sense if we're gonna combat breach of data.*
Transparency	… there is clear communication about how the wGT program works—the process and data protections.	ELSI stakeholder: *The employees are not gonna see the terms of that contract. They're not gonna know what the data agreements necessarily are and the details about the stated purposes of those data. There might be something that's—like a notice that's given to people, the employees who might be participating there that says, "We're gonna aggregate data. Any data would be aggregated or deidentified and shared with the employer and won't be used against you," or something like that.*<br>Employee stakeholder: *I think information on what's involved … the information on what GINA is all about and how it affects the use of my data would at least make me feel better about the genetic testing.*
Understanding/education	… employees have a good understanding of wGT before and after the test (for example, education, genetic counseling).	Genetic testing industry stakeholder: *I'll just say I support access to [wGT]. I support employers wanting to institute these programs as long as they have the right educational programs in place so that patients understand the kinds of information that they're getting, and what they may or may not be able to do with that information.*<br>ELSI stakeholder: *I think that there needs to be good education around whatever is being offered. I think it's also hard because most of this is sort of in a direct-to-consumer way. I'm assuming they don't always require genetic counseling … probably because it would make it more expensive. Especially for people who are not genetics experts taking these tests, there could be misunderstanding of what the information is telling them or false reassurance if they have a negative result or nothing is identified. I think there's a danger in misunderstanding and then feeling safe from whatever it is that you didn't have … I think that is definitely a risk that would be hard to get around. 'Cause even if you're providing the education, people don't always pay attention to that. I skip a lot of stuff that I should be reading and just, "Okay. That looks good. I'll sign it." I think that's a risk.*

(*Continued on next page*)

Table 3. *Continued*

Key characteristic	Description: Measures to ensure …	Representative quote(s)
User-friendliness	… the wGT program has a simple and easy-to-use design.	Healthcare professional stakeholder: *In addition to the option to have the in-person consultative telegenetics type of consultative service offering information in different formats and modalities … it'd be great to have a video somebody could just click and see and get some information. Just understanding people getting information in different ways or receive that information in different ways. They may not have time to sit down and have a counseling session. Maybe they can listen to a video in their car while they're riding.*<br>Employee stakeholder: *I don't know if this falls under user friendliness or not, but how is the test actually administered? Because I think it's one thing if it's just you put a piece of your hair in an envelope and you mail it back in … Or is it something where you have to do a blood draw or a mouth swab? If so, is your workplace gonna offer that test at the workplace? Are you gonna be given release time to go and get this done? Are you expected to do it on your own time?*
Utility for employers	… employers can reap benefits through offering wGT to their employees.	ELSI stakeholder: *Employees may appreciate the idea that their employer cares about them and their health and thinks—people like getting, quote, perks. To the extent that people feel better about their employer and their employment as a result of having this option, that could be a benefit to employees as well as employers.*<br>Employee stakeholder: *Maybe the assumption is that … [with wGT], maybe you're gonna miss less sick leaves or PTO or what-not and work on, I don't know, a lot of preventative measures and be at work and be more productive, and your mood is better. I don't know. Maybe for the long run, maybe that'll reduce employee costs, and they'll also be alive. It's one less person to hire.*<br>Genetic testing industry stakeholder: *… the primary benefit for the employers is … to be able to appear to be very progressive and concerned and cutting edge, and to attract certain employers who may be of a particular mindset … they're using it primarily as a competitive advantage …*
Utility for laboratories	… laboratories can reap the benefits of performing and/or offering services related to wGT.	Healthcare professional stakeholder: *For me, it's a free market system. Vendors are seeing a market and an opportunity … to make a business plan or a business model out of offering [wGT].*<br>ELSI stakeholder: *It's in [vendors'] interest to emphasize the purported benefits of testing of all sorts, and I would assume that genetic testing would fall into that broad category.*
Voluntariness	… prevention of coercion or pressure to participate in wGT.	Genetic testing industry stakeholder: *I wanted to touch on the voluntariness, lack of coercion. To me that is not a design feature, that's a requirement … if you can't even offer it without coercing individuals, then … there's no way to ever offer it.*<br>Employee stakeholder: *I, also, would make voluntariness or lack of coercion a must-have. Requiring genetic testing is a little much for me. It's a little 1984 or somethin' like that. Think it has to be voluntary.*

additional details about participant endorsement of importance, likelihood of being achieved, and necessity of the 12 key characteristics for wGT.

Evaluating stakeholder acceptance of wGT

The majority of participants were categorized as either supportive ($n = 10$ of 30, 33%) or libertarian ($n = 8$ of 30, 27%) regarding wGT based on their Likert-scale responses on Survey 1 (Table 4). By Survey 2, after the Deliberative Workshop, most participants had shifted to either permissive ($n = 12$ of 30, 40%) or opposed ($n = 8$ of 30, 27%) to wGT, indicating increased skepticism. In Survey 2, when assuming that the key characteristics they deemed "necessary" for wGT to be offered were achieved, most participants were either categorized as supportive ($n = 11$ of 30, 37%) or permissive ($n = 9$ of 30, 30%) of wGT (Table 4). While it appeared that individuals in the employee and genetic testing industry stakeholder groups began the study slightly more supportive or permissive than others, major differences in acceptance of wGT by stakeholder group were not clearly observed, both in Survey 1 and throughout the study.

Quantitative analysis supported descriptive trends. Results of chi-square analysis revealed participants were significantly more likely to move toward agreement that wGT should NOT be allowed after the Deliberative Workshop, between Survey 1 and Survey 2 ($p = 0.02$). However, from the beginning to the end of Survey 2, participants were significantly more likely to move toward (1) disagreement that wGT should NOT be allowed ($p = 0.02$) and (2) agreement that wGT should be offered ($p = 0.01$), assuming their key characteristics were met (Table 5).

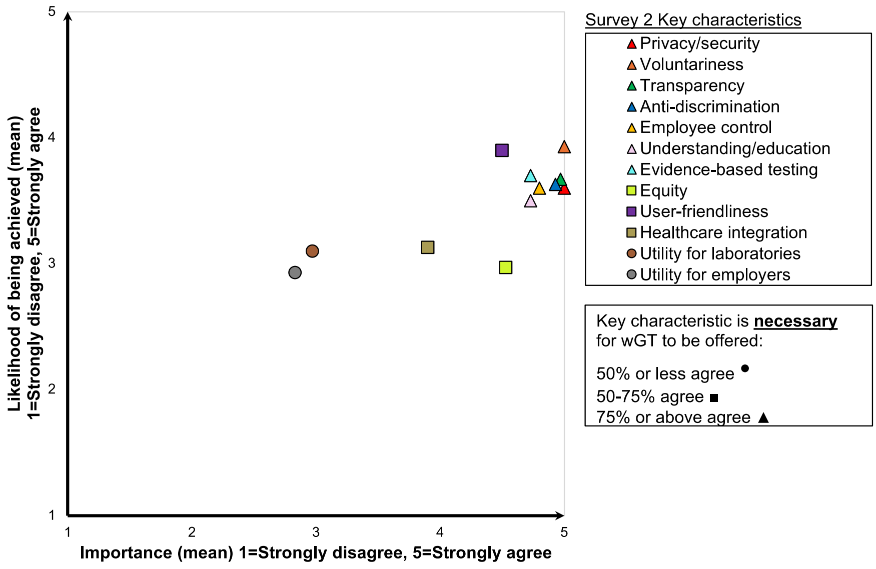

Figure 1. Key characteristics of workplace genetic testing (wGT) and their importance, necessity, and likelihood of being achieved as perceived by participants ($n = 30$)
Importance (x), likelihood of being achieved (y), with shapes of symbols (legend) denoting the percentage of participants who indicated the key characteristic was "necessary" for wGT to be offered. Values plotted on the x/y axes represent mean of Likert-scale scores for each characteristic.

Discussion

We conducted a modified Delphi process with workplace genetic testing stakeholders to identify and assess key characteristics for implementation of wGT that could maximize benefits and minimize harms, and to explore stakeholder acceptance. Twelve key characteristics emerged: employee control of data, equity in access, evidence-based testing, healthcare integration, programmatic transparency, user-friendliness, utility for employers, utility for laboratories, pre- and post-test education, voluntariness of participation, anti-discrimination measures, and the privacy and security of information (Table 3). The latter four mirror many of the top ethical, legal, and social criteria identified in previous studies on dtcGT.[42–44] The identified key characteristics also align well with accepted ethical principles in clinical care delivery,[53] research ethics,[54] and public health practice.[55] Programmatic design features that supported employee data control were reported to increase employee participation in a hypothetical wGT program in one recent study,[16] which is also consistent with our findings. While many of these key characteristics are not unique to wGT, and may broadly apply to genetic and genomic testing, the implications for achieving them require careful consideration of the employee-employer relationship. For example, in order for privacy and security of genomic data to be achieved in a wGT program, one must consider the employer's potential access to data and the relevant laws, as GINA currently explicitly allows employers to receive aggregate employee data on wGT.[6,56]

Most participants agreed that these key characteristics were important and necessary for implementation of wGT. However, some participants expressed doubts about the likelihood of achieving all key characteristics due to structural challenges, such as ensuring equitable access to genetic and healthcare services for all employees, incorporating genetic testing data into electronic health record systems, and providing adequate genomics education for healthcare providers. These findings raise questions about the feasibility of implementing wGT in adherence to stakeholders' ethical values and preferences. Implementation studies may help to further clarify the feasibility of incorporating such elements in wGT program design.

These findings may also point to an overall, underlying lack of trust in employers and genetic testing vendors in general to ensure employee privacy, security, and nondiscrimination. Prior to the introduction of GINA, the potential for overt genetic discrimination in the workplace was a concern. For example, in *Norman-Bloodsaw v. Lawrence Berkeley Labs* (1998), an employer was sued for testing Black employees for sickle cell carrier status without their knowledge.[6,57] Although GINA now provides some genetic privacy protections, including protections against genetic discrimination in employment, studies have shown that the public lacks awareness of GINA, and its existence does not fully alleviate concerns about potential genetic discrimination.[10,32,58] Other recent events, such as 23andMe's data breach and bankruptcy announcement have shed light onto the challenges of trusting private companies, outside the healthcare arena, with sensitive genetic data.[59–61]

Overall, participants' responses to questions measuring their acceptance of wGT demonstrated they had qualified support for wGT being offered, conditional on whether all characteristics that individuals felt were "necessary" could be met. These findings may suggest that wGT could be a viable model for offering genetic testing. However, ensuring that key characteristics are achieved would require substantial policy development and significant changes to infrastructure and processes within companies, genetic testing laboratories, regulatory bodies, and healthcare systems. For example, since stakeholders expressed concerns about the feasibility of integrating wGT into existing healthcare systems, it would be necessary to develop effective methods for ensuring that genetic test results from wGT can be easily and securely shared with healthcare providers via electronic health

Table 4. Shifts in stakeholder acceptance of workplace genetic testing (wGT) before and after the deliberative workshop based on quantitative and open-ended survey responses ($n = 30$)

Category[a]	Survey 1[b] N (%)	Survey 2[b] N (%)	Survey 2, assuming key characteristics met[b] N (%)	Exemplary quote
Supportive	10 (33)	4 (13)	11 (37)	*Given the types of benefits currently being offered by employers, I think genetic testing fits in well and is a compelling benefit, especially as genetic testing accessibility is so limited and is not being provided even to those who meet criteria and should be getting it. This provides the opportunity for access to more people.*
Permissive	4 (13)	12 (40)	9 (30)	*I believe that if the employer determines that there is substantial value to the employee and, by extension, to the organization in offering the testing, the employer should be allowed to do so. I do not believe that employers have an obligation to offer this service, but neither do I feel that they should be discouraged from doing so. In essence, I find no absolute imperative to pursue either course of action.*
Neutral/Conflicted	6 (20)	3 (10)	1 (3)	*It depends on the safeguards in place.*
Libertarian	8 (27)	3 (10)	5 (17)	*I can't support any measure to prevent what an employer offers as an employee option. The marketplace should determine an employer's benefit programs.*
Opposed	2 (7)	8 (27)	4 (13)	*… I believe the workplace should keep their grubby, greedy hands off of my genetics.*

[a]Participants perspectives on whether wGT should (1) be offered, and (2) not be allowed, from Survey 1, to Survey 2, to Survey 2 assuming the 12 key characteristics are met. Categories based on levels of support or opposition to wGT were assigned to groups of participants based on quantitative survey responses and corresponding exemplary quotes (supportive, permissive, neutral/conflicted, libertarian, or opposed; see methods, Table 1 for additional details).
[b]The Deliberative Workshop took place between Survey 1 and Survey 2.

records, with employees' consent. Additionally, given employee data control was deemed very important by most stakeholders, it might be important to consider how employees would be able to withdraw their wGT-related data, including deidentified data from a laboratory's database, which may require organizational policy and legal changes related to laboratory record retention. These key characteristics should be considered as workplace genetic testing continues to be implemented,[62] and as workplace wellness programs involving genetic testing evolve.[63]

Strengths and limitations

Our study is one of the first to systematically define key characteristics for wGT that, if achieved, could maximize potential benefits while reducing risks of harm. A strength of the study is that the findings are derived from a diverse group of stakeholders, including those who are potentially involved in or directly affected by wGT. The design of the modified Delphi process combined with a Deliberative Workshop ensured that stakeholders of varying levels of genetic expertise could have their perspectives heard and validated in the development of the key characteristics. However, the generalizability of our findings is likely limited by the relatively small number of participants from each stakeholder group. Furthermore, despite our efforts to recruit for diversity, the diversity within our study population was not as high as desired. Additional national studies investigating stakeholder perspectives[9,10] are needed to develop a more comprehensive understanding of wGT stakeholder viewpoints.

We also observed a 30% attrition rate, primarily among healthcare professionals and employers, while 100% of genetic testing industry representatives finished all steps of the study, possibly biasing our results toward the opinions of this group. However, the attrition rate was relatively small compared with other multi-step Delphi processes that primarily include surveys.[64] Our modified Delphi process required more of participants, who were asked to engage in a 5-h deliberative workshop.

We specifically chose dichotomous questions to evaluate stakeholder acceptance of wGT, which included one potentially confusing negative statement (e.g., "Employers should NOT be allowed"). We observed apparent contradictions in how participants answered these questions (e.g., three quantitative survey responses did not seem to match the associated qualitative response) and received feedback from participants during the workshop that the dichotomous format was challenging. Nevertheless, framing the questions as a dichotomous choice allowed us to track changes in participants' perspectives over time and to determine whether the key characteristics would influence their ultimate acceptance of wGT.

Conclusions

This study presents the results of the first modified Delphi process to explore important stakeholder perspectives in order to identify key characteristics for maximizing

Table 5. Shifts in stakeholder acceptance of workplace genetic testing (wGT) before and after the Deliberative Workshop based on quantitative survey responses and chi-square analysis ($n = 30$)

Survey question[a] (timing of survey)	No change in agreement N (%)	Shifted toward disagree N (%)	Shifted toward agree N (%)	P value[b]
**Q1. Employers should NOT be allowed to offer wGT[c]**				
(Survey 1 vs. Survey 2)	16 (53)	3 (10)	11 (37)	0.02
(Survey 1 vs. Survey 2, Assuming key characteristics met)	16 (53)	7 (23)	7 (23)	1.0
(Survey 2 vs. Survey 2, Assuming key characteristics met)	22 (73)	7 (23)	1 (3)	0.02
**Q2. Employers should offer wGT[c]**				
(Survey 1 vs. Survey 2)	15 (50)	10 (33)	5 (17)	0.14
(Survey 1 vs. Survey 2, Assuming key characteristics met)	14 (47)	7 (23)	9 (30)	0.56
(Survey 2 vs. Survey 2, Assuming key characteristics met)	18 (60)	2 (7)	10 (33)	0.01

[a]Participants were asked to rate their agreement with Q1 and Q2 in both Survey 1 and Survey 2. The Deliberative Workshop took place between Survey 1 and Survey 2. Later in Survey 2, participants were again asked to rate their agreement with these items, assuming the key characteristics for wGT programs that they described as "necessary" were met.
[b]Chi-square analysis was used to compare the percentage of participants who shifted toward *disagreement* with the percentage who shifted toward *agreement* with Q1 and Q2 before and after the Deliberative Workshop.
[c]Likert-scale question: Strongly agree to strongly disagree.

benefit and reducing potential harm in the implementation of wGT. The characteristics prioritized by stakeholders should be considered in the programmatic design of wGT. However, given stakeholders' lack of confidence in achieving some of the proposed key characteristics in the context of wGT, further evaluation of their feasibility is needed. These findings help to inform the development of a normative framework for wGT.

Data and code availability

The survey instruments are available in the supplemental materials of this article. Other study materials and quantitative data that support the findings of this study are available at Inter-university Consortium for Political and Social Research (ICPSR) based at the University of Michigan under ICPSR-229864. The raw qualitative data are not publicly available due to privacy or ethical restrictions.

Consortia

The INSIGHT @ Work consortium includes the following core team members, in addition to the named authors: Betty Cohn, MBE, Nicole Crumpler, MS, MBA, Rebecca Ferber, MPH, Veda N. Giri, MD, Katherine Hendy, MA, PhD, Amy Leader, MPH, DrPH, Debra Mathews, MA, PhD, Sarah McCain, MPH, Kayte Spector-Bagdady, MBE, JD, and Wendy R. Uhlmann, MS, CGC. The consortium is supported by advisory board members Kyle Brothers, Ellen Wright Clayton, Patricia Deverka, Thomas Ellis, Aaron Goldenberg, Susan Mockus, Cynthia Casson Morton, Jens Rueter, and Brett Witham, along with stakeholder workgroup members Ethan Bessey, Erynn Gordon, LaTasha Lee, Jessica Roberts, and Fatima Saidi

Acknowledgments

This research was supported by a grant from the National Human Genome Research Institute of the National Institutes of Health (R01HG010679). The content is solely the responsibility of the authors and does not necessarily represent the official views of the National Institutes of Health.

Author contributions

First author E.C. led the writing of the original draft, curated all data, and contributed to formal analysis, methodology, investigation, and project administration with supervision from K.S. Senior authors A.E.R.P. and W.G.F. led the investigation and methodology, provided supervision, and contributed to formal analysis and project administration. E.C., K.S., K.A.R., A.V., A. T., A.E.R.P., and W.G.F. contributed to conceptualization, investigation, data curation, and project administration. K.A.R. provided additional expertise regarding methodology and contributed to formal analysis. K.S., K.A.R., A.T., A.E.R.P., and W.G.F. also contributed to the original draft and provided supervision during the writing process. S.P. and J.M.R. provided software and conducted inferential statistical analysis. K.S., C.L., and J.S. R. were responsible for funding acquisition. C.L. and J.S.R. provided oversight of all research and administrative activities as multiple principal investigators. All authors read and approved the final manuscript.

Declaration of interests

The authors declare no competing interests.

Supplemental information

Supplemental information can be found online at https://doi.org/10.1016/j.xhgg.2025.100458.

Received: January 23, 2025
Accepted: May 15, 2025

References

1. Halbisen, A.L., and Lu, C.Y. (2023). Trends in Availability of Genetic Tests in the United States, 2012-2022. J. Pers. Med. *13*, 638. https://doi.org/10.3390/jpm13040638.
2. Dusic, E.J., Theoryn, T., Wang, C., Swisher, E.M., Bowen, D.J.; and EDGE Study Team (2022). Barriers, interventions, and recommendations: Improving the genetic testing landscape. Front. Digit. Health *4*, 961128. https://doi.org/10.3389/fdgth.2022.961128.
3. Oh, B. (2019). Direct-to-consumer genetic testing: advantages and pitfalls. Genomics Inform. *17*, e33. https://doi.org/10.5808/GI.2019.17.3.e33.
4. Helgason, A., and Stefánsson, K. (2010). The past, present, and future of direct-to-consumer genetic tests. Dialogues Clin. Neurosci. *12*, 61–68. https://doi.org/10.31887/DCNS.2010.12.1/ahelgason.
5. Kirby, H.G., Rehm, H.L., and Hull, L.E. (2023). An Environmental Scan of Consumer-Initiated Germline Genetic Testing for Health Risks. Mayo Clin. Proc. *98*, 1529–1543. https://doi.org/10.1016/j.mayocp.2023.04.008.
6. Sanghavi, K., Cohn, B., Prince, A.E.R., Feero, W.G., Ryan, K. A., Spector-Bagdady, K., Uhlmann, W.R., Lee, C., Roberts, J. S., and Mathews, D.J.H. (2022). Voluntary workplace genomic testing: wellness benefit or Pandora's box? NPJ Genom. Med. *7*, 5. https://doi.org/10.1038/s41525-021-00276-8.
7. Briscoe, F., Ajunwa, I., Bourgoin, A., and Maxwell, J. (2023). Racial Differences in Perceptions of Genetic Wellness Programs. Am. J. Health Promot. *37*, 940–952. https://doi.org/10.1177/08901171231184360.
8. Cohn, I., Manshaei, R., Liston, E., Okello, J.B.A., Khan, R., Curtis, M.R., Krupski, A.J., Jobling, R.K., Kalbfleisch, K., Paton, T.A., et al. (2021). Assessment of the Implementation of Pharmacogenomic Testing in a Pediatric Tertiary Care Setting. JAMA Netw. Open *4*, e2110446. https://doi.org/10.1001/jamanetworkopen.2021.10446.
9. Charnysh, E., Pal, S., Reader, J.M., Uhlmann, W.R., McCain, S., Sanghavi, K., Blasco, D., Brandt, R., Feero, W.G., Ferber, R., et al. (2024). Health care utilization and behavior changes after workplace genetic testing at a large US health care system. Genet. Med. *26*, 101160. https://doi.org/10.1016/j.gim.2024.101160.
10. Willard, L., Uhlmann, W., Prince, A.E.R., Blasco, D., Pal, S., Roberts, J.S.; and INSIGHT @ Work Consortium (2025). The Genetic Information Nondiscrimination Act and workplace genetic testing: Knowledge and perceptions of employed adults in the United States. J. Genet. Couns. *34*, e1945. https://doi.org/10.1002/jgc4.1945.
11. Cohn, B., Ryan, K.A., Hendy, K., Callahan, K., Roberts, J.S., Spector-Bagdady, K., Mathews, D.J.H.; and INSIGHT @ Work Consortium (2023). Genomic testing in voluntary workplace wellness programs in the US: Evidence and challenges. Mol. Genet. Genomic Med. *11*, e2245. https://doi.org/10.1002/mgg3.2245.
12. M. Over, G. Memberships. The Hot New Employee Perk Involves Spitting Into a Cup. (2018).
13. Employees Jump at Genetic Testing. Is That a Good Thing? (2018). Published online April 15, 2018.
14. Jefferson to Offer Free Genetic Testing to Employees. (2018). Published online November 14, 2018.
15. Sanghavi, K., Feero, W.G., Mathews, D.J.H., Prince, A.E.R., Price, L.L., Liu, E.T., Brothers, K.B., Roberts, J.S., and Lee, C. (2021). Employees' Views and Ethical, Legal, and Social Implications Assessment of Voluntary Workplace Genomic Testing. Front. Genet. *12*, 643304. https://doi.org/10.3389/fgene.2021.643304.
16. Briscoe, F., Maxwell, J.H., and Bourgoin, A. (2024). Workplace genetic testing: which employees are likely to participate, what are their concerns with employer sponsorship, and which design features could reduce barriers and increase participation? Front. Genet. *15*, 1496900. https://doi.org/10.3389/fgene.2024.1496900.
17. Roberts, J.S., and Ostergren, J. (2013). Direct-to-Consumer Genetic Testing and Personal Genomics Services: A Review of Recent Empirical Studies. Curr. Genet. Med. Rep. *1*, 182–200. https://doi.org/10.1007/s40142-013-0018-2.
18. Martins, M.F., Murry, L.T., Telford, L., and Moriarty, F. (2022). Direct-to-consumer genetic testing: an updated systematic review of healthcare professionals' knowledge and views, and ethical and legal concerns. Eur. J. Hum. Genet. *30*, 1331–1343. https://doi.org/10.1038/s41431-022-01205-8.
19. Jiang, S., Liberti, L., and Lebo, D. (2023). Direct-to-Consumer Genetic Testing: A Comprehensive Review. Ther. Innov. Regul. Sci. *57*, 1190–1198. https://doi.org/10.1007/s43441-023-00567-5.
20. Public Perspectives on Personalized Medicine: A Survey of U. S. Public Opinion. (2018). Personalized Medicine Coalition. https://www.personalizedmedicinecoalition.org/Userfiles/PMC-Corporate/file/Public_Perspectives_on_PM1.pdf.
21. Apathy, N.C., Menser, T., Keeran, L.M., Ford, E.W., Harle, C. A., and Huerta, T.R. (2018). Trends and Gaps in Awareness of Direct-to-Consumer Genetic Tests From 2007 to 2014. Am. J. Prev. Med. *54*, 806–813. https://doi.org/10.1016/j.amepre.2018.02.013.
22. Salloum, R.G., George, T.J., Silver, N., Markham, M.J., Hall, J. M., Guo, Y., Bian, J., and Shenkman, E.A. (2018). Rural-urban and racial-ethnic differences in awareness of direct-to-consumer genetic testing. BMC Public Health *18*, 277. https://doi.org/10.1186/s12889-018-5190-6.
23. Friedman, A., and Anderson, T.L. (2024). Motivations for Direct-to-Consumer Genetic Testing: Understanding Interpretations of Ancestry Results. Qual. Sociol. *47*, 543–569. https://doi.org/10.1007/s11133-024-09569-7.
24. Hsieh, V., Braid, T., Gordon, E., and Hercher, L. (2021). Direct-to-consumer genetic testing companies tell their customers to 'see a genetic counselor'. How do genetic counselors feel about direct-to-consumer genetic testing? J. Genet. Couns. *30*, 191–197. https://doi.org/10.1002/jgc4.1310.
25. Middleton, A., Mendes, Á., Benjamin, C.M., and Howard, H. C. (2017). Direct-to-consumer genetic testing: where and how does genetic counseling fit? Per. Med. *14*, 249–257. https://doi.org/10.2217/pme-2017-0001.
26. Bloss, C.S., Ornowski, L., Silver, E., Cargill, M., Vanier, V., Schork, N.J., and Topol, E.J. (2010). Consumer perceptions of direct-to-consumer personalized genomic risk assessments. Genet. Med. *12*, 556–566. https://doi.org/10.1097/GIM.0b013e3181eb51c6.
27. Hingorani, A.D., Gratton, J., Finan, C., Schmidt, A.F., Patel, R., Sofat, R., Kuan, V., Langenberg, C., Hemingway, H., Morris, J.

K., and Wald, N.J. (2023). Performance of polygenic risk scores in screening, prediction, and risk stratification: secondary analysis of data in the Polygenic Score Catalog. BMJ Med. *2*, e000554. https://doi.org/10.1136/bmjmed-2023-000554.
28. Stoekle, H.C., Mamzer-Bruneel, M.F., Vogt, G., and Herve, C. (2016). 23andMe: a new two-sided data-banking market model. BMC Med. Ethics *17*, 19. https://doi.org/10.1186/s12910-016-0101-9.
29. Bernhardt, B.A., Zayac, C., Gordon, E.S., Wawak, L., Pyeritz, R.E., and Gollust, S.E. (2012). Incorporating direct-to-consumer genomic information into patient care: attitudes and experiences of primary care physicians. Per. Med. *9*, 683–692. https://doi.org/10.2217/pme.12.80.
30. Mladucky, J., Baty, B., Botkin, J., and Anderson, R. (2021). Secondary Data Usage in Direct-to-Consumer Genetic Testing: To What Extent Are Customers Aware and Concerned? Public Health Genom. *24*, 199–206. https://doi.org/10.1159/000512660.
31. Ziegler, E., Mladucky, J., Baty, B., Anderson, R., and Botkin, J. (2022). Knowledge and Attitudes about Privacy and Secondary Data Use among African-Americans Using Direct-to-Consumer Genetic Testing. Public Health Genom. *25*, 164–173. https://doi.org/10.1159/000525902.
32. Raz, A.E., Niemiec, E., Howard, H.C., Sterckx, S., Cockbain, J., and Prainsack, B. (2020). Transparency, consent and trust in the use of customers' data by an online genetic testing company: an Exploratory survey among 23andMe users. New Genet. Soc. *39*, 459–482. https://doi.org/10.1080/14636778.2020.1755636.
33. Mujtaba, B.G., and Cavico, F.J. (2013). Corporate wellness programs: implementation challenges in the modern american workplace. Int. J. Health Policy Manag. *1*, 193–199. https://doi.org/10.15171/ijhpm.2013.36.
34. Goetzel, R.Z., Henke, R.M., Tabrizi, M., Pelletier, K.R., Loeppke, R., Ballard, D.W., Grossmeier, J., Anderson, D.R., Yach, D., Kelly, R.K., et al. (2014). Do workplace health promotion (wellness) programs work? J. Occup. Environ. Med. *56*, 927–934. https://doi.org/10.1097/JOM.0000000000000276.
35. Song, Z., and Baicker, K. (2019). Effect of a Workplace Wellness Program on Employee Health and Economic Outcomes: A Randomized Clinical Trial. JAMA *321*, 1491–1501. https://doi.org/10.1001/jama.2019.3307.
36. Jones, D., Molitor, D., and Reif, J. (2019). What do Workplace Wellness Programs do? Evidence from the Illinois Workplace Wellness Study. Q. J. Econ. *134*, 1747–1791. https://doi.org/10.1093/qje/qjz023.
37. Strassle, E., and Berkman, B.E. (2020). Workplace Wellness Programs: Empirical Doubt, Legal Amiguity, and Conceptual Confusion. William Mary Law Rev. *61*, 1666.
38. Perrault, E.K., Hildenbrand, G.M., and Rnoh, R.H. (2020). Employees' Refusals to Participate in an Employer-Sponsored Wellness Program: Barriers and Benefits to Engagement. Compensat. Benefit Rev. *52*, 8–18. https://doi.org/10.1177/0886368719899209.
39. Fleming, H.K. (2020). Navigating Workplace Wellness Programs in the Age of Technology and Big Data. J. Sci. Policy Gov., 17. https://doi.org/10.38126/JSPG170104.
40. Pomeranz, J.L. (2015). Participatory workplace wellness programs: reward, penalty, and regulatory conflict. Milbank Q. *93*, 301–318. https://doi.org/10.1111/1468-0009.12123.
41. Horwitz, J.R., Kelly, B.D., and DiNardo, J.E. (2013). Wellness incentives in the workplace: cost savings through cost shifting to unhealthy workers. Health Aff. *32*, 468–476. https://doi.org/10.1377/hlthaff.2012.0683.
42. Ascencio-Carbajal, T., Saruwatari-Zavala, G., Navarro-Garcia, F., and Frixione, E. (2021). Genetic/genomic testing: defining the parameters for ethical, legal and social implications (ELSI). BMC Med. Ethics *22*, 156. https://doi.org/10.1186/s12910-021-00720-5.
43. Cernat, A., Bashir, N.S., and Ungar, W.J. (2022). Considerations for developing regulations for direct-to-consumer genetic testing: a scoping review using the 3-I framework. J. Community Genet. *13*, 155–170. https://doi.org/10.1007/s12687-022-00582-3.
44. Onstwedder, S.M., Jansen, M.E., Cornel, M.C., and Rigter, T. (2024). Policy Guidance for Direct-to-Consumer Genetic Testing Services: Framework Development Study. J. Med. Internet Res. *26*, e47389. https://doi.org/10.2196/47389.
45. Majumder, M.A., Blank, M.L., Geary, J., Bollinger, J.M., Guerrini, C.J., Robinson, J.O., Canfield, I., Cook-Deegan, R., and McGuire, A.L. (2021). Challenges to Building a Gene Variant Commons to Assess Hereditary Cancer Risk: Results of a Modified Policy Delphi Panel Deliberation. J. Pers. Med. *11*, 646. https://doi.org/10.3390/jpm11070646.
46. Ryan, K.A., Cohen-Mekelburg, S., Baker, J.A., Weinheimer-Haus, E.M., Krenz, C., Hou, J.K., De Vries, R., and Waljee, A. K. (2024). Public deliberation to assess patient views on biosimilar medication switching for the treatment of inflammatory bowel disease. BMC Health Serv. Res. *24*, 1209. https://doi.org/10.1186/s12913-024-11570-3.
47. Raj, M., Ryan, K., Nong, P., Calhoun, K., Trinidad, M.G., De Vries, R., Creary, M., Spector-Bagdady, K., Kardia, S.L.R., and Platt, J. (2022). Public Deliberation Process on Patient Perspectives on Health Information Sharing: Evaluative Descriptive Study. JMIR Cancer *8*, e37793. https://doi.org/10.2196/37793.
48. Fitch, K. (2001). The Rand/UCLA appropriateness method user's manual (Rand).
49. Hsu, C., and Sandford, B.A. (2007). The Delphi Technique: Making Sense of Consensus. Practical Assessment, Research and Evaluation *12*. https://doi.org/10.7275/pdz9-th90.
50. McMillan, S.S., King, M., and Tully, M.P. (2016). How to use the nominal group and Delphi techniques. Int. J. Clin. Pharm. *38*, 655–662. https://doi.org/10.1007/s11096-016-0257-x.
51. Nair, R., Aggarwal, R., and Khanna, D. (2011). Methods of formal consensus in classification/diagnostic criteria and guideline development. Semin. Arthritis Rheum. *41*, 95–105. https://doi.org/10.1016/j.semarthrit.2010.12.001.
52. Amini, K., Buscetta, A.J., Ramirez, H.C., and Bonham, V.L. (2022). Democratizing Knowledge for Sickle Cell Disease Gene Therapy: A Community Based Model for Creating Patient Education Materials. Blood *140*, 7871–7872. https://doi.org/10.1182/blood-2022-169181.
53. AMA Code of Medical Ethics: Principles of Medical Ethics. (2001). https://code-medical-ethics.ama-assn.org/principles.
54. Miracle, V.A. (2016). The Belmont Report: The Triple Crown of Research Ethics. Dimens. Crit. Care Nurs. *35*, 223–228. https://doi.org/10.1097/DCC.0000000000000186.
55. (2019). Public Health Code of Ethics (American Public Health Association).
56. Genetic Information Nondiscrimination Act. (2008). H. R. 493.

57. U S Court of Appeals Ninth Circuit (1998). Norman-Bloodsaw v. Lawrence Berkeley Laboratory. Fed. Report. *135*, 1260–1276.
58. Lenartz, A., Scherer, A.M., Uhlmann, W.R., Suter, S.M., Anderson Hartley, C., and Prince, A.E.R. (2021). The persistent lack of knowledge and misunderstanding of the Genetic Information Nondiscrimination Act (GINA) more than a decade after passage. Genet. Med. *23*, 2324–2334. https://doi.org/10.1038/s41436-021-01268-w.
59. Prince, A.E.R., and Spector-Bagdady, K. (2025). Protecting Privacy When Genetic Databases Are Commercialized. JAMA *333*, 665–666. https://doi.org/10.1001/jama.2024.26279.
60. What went wrong at 23andMe? Why the genetic-data giant risks collapse. (2025).
61. What happens to your DNA now that 23andMe has filed for bankruptcy? (2025).
62. MyOme, Novi Health Bring Whole-Genome Testing to Workplace Health Programs. (2025).
63. Color Health builds out virtual cancer clinic, taking aim at employer, health plan market. (2024).
64. Shang, Z. (2023). Use of Delphi in health sciences research: A narrative review. Medicine (Baltim.) *102*, e32829. https://doi.org/10.1097/MD.0000000000032829.

HGGA, Volume *6*

Supplemental information

Multidisciplinary stakeholder-informed identification of key characteristics for implementation of workplace genetic testing

Elizabeth Charnysh, Kunal Sanghavi, Kerry A. Ryan, Alyx Vogle, Alexandra Truhlar, Subhamoy Pal, Jonathan M. Reader, J. Scott Roberts, Charles Lee, Anya E.R. Prince, W. Gregory Feero, and INSIGHT @ Work Consortium

Supplemental Materials

SURVEY INSTRUMENTS

SURVEY 1

Demographics Please fill out the following demographic characteristics so we may appropriately understand your role as stakeholder in this deliberative process. **Click the arrow below to continue.**

Start of Block: Demographics

What are your stakeholder role(s)/perspectives with regard to the genetic testing wellness programs? Please select your **primary perspective** for this process.

- ○ Employee organization (e.g., union leader, employee advocacy group)
- ○ Employer or human resources
- ○ Ethical, legal, and social implications or bioethics
- ○ Genetic counselor or other healthcare professional
- ○ Genetic testing company
- ○ Government regulator (e.g., EEOC, HHS)
- ○ Insurance company
- ○ Privacy and/or data protection
- ○ Employee
- ○ Other (please specify): ____

Please briefly describe your current or most recent role in this field.

____

____

____

____

____

How many years have you been in this field?

- ○ Less than 1 year
- ○ 1-4 years
- ○ 5-10 years
- ○ 11-15 years
- ○ More than 15 years

Page Break

What is your highest level of education?

- ○ Some high school
- ○ High school graduate (diploma or GED certificate)
- ○ Some college, no degree (includes some community college)
- ○ Two-year associate degree from a college or university
- ○ Technical/trade school or apprenticeship (e.g., electrician, plumbing, culinary school)
- ○ Four-year college or university degree/Bachelor’s degree (e.g., BS, BA, AB)
- ○ Some postgraduate or professional schooling, no postgraduate degree (e.g., some graduate school)
- ○ Postgraduate or professional degree, including master's, doctorate, medical or law degree (e.g., MA, MS, PhD, JD, graduate school)

What is your age?

____

End of Block: Demographics

Start of Block: Delphi Round 1 Intro

Delphi Survey: Round 1
As part of a workplace wellness program, a number of US employers are offering their employees workplace genetic testing (wGT). We are convening a deliberative process including a workshop with key stakeholders (including employees, employers, labor organization representatives, ethicists, insurance companies, genetic testing companies, health care providers, policy makers and legal experts) to identify the ethical, legal, social, and policy implications of wGT and to suggest practice and policy options to guide implementation and governance of such testing. Please complete this 20 minute survey that will help inform the workshop. **Click the arrow below to continue.**

End of Block: Delphi Round 1 Intro

Start of Block: Block 9

Please watch the less-than-5-minute video below before proceeding to learn important background information about the research study.

The survey includes a short video. You will need to play it with sound. If you are not able to have your sound on at this time, feel free to take this survey later when you can play the sound. If you will not be able to hear the video, click on the CC button to enable closed captioning.

https://youtu.be/kyTCELnP3Bs

End of Block: Block 9

Start of Block: Survey Part 1

Perspectives on workplace genetic testing Please review the definition of workplace genetic testing before continuing.

Workplace genetic testing: Voluntary genetic testing offered to employees as part of a workplace wellness program. This testing is not initiated by a provider and is not typically discussed in a visit with their clinician prior to testing. For example, a company may offer their employees the option to pursue genetic testing for inherited conditions that increase someone's risk for cancer or heart disease.

For each of the next 11 questions, please describe your answers in detail and give examples as needed. There are no right or wrong answers; we want to know your experiences, views, and perceptions. You will be given an opportunity to provide possible benefits and risks of workplace wellness genetic testing.
Click the arrow below to continue.

End of Block: Survey Part 1

Start of Block: Benefits

Please describe **up to five** potential **benefits** of workplace wellness genetic testing.

○ ____

○ ____

○ ____

○ ____

○ ____

Workplace genetic testing: Voluntary genetic testing offered to employees as part of a workplace wellness program. This testing is not initiated by a provider and is not typically discussed in a visit with their clinician prior to testing. For example, a company may offer their employees the option to pursue genetic testing for inherited conditions that increase someone's risk for cancer or heart disease.

Carry Forward Entered Choices - Entered Text from "Please describe up to five potential benefits of workplace wellness genetic testing."

Please click and drag to rank **up to five** potential **benefits** of workplace wellness genetic testing.

______

______

______

______

______

Please describe any other potential **benefits** of workplace wellness genetic testing.

____

____

____

____

End of Block: Benefits

Start of Block: Risks/Harms

Please describe **up to five** potential **risks/harms** of workplace wellness genetic testing.

○ ____

○ ____

○ ____

○ ____

○ ____

Workplace genetic testing: Voluntary genetic testing offered to employees as part of a workplace wellness program. This testing is not initiated by a provider and is not typically discussed in a visit with their clinician prior to testing. For example, a company may offer their employees the option to pursue genetic testing for inherited conditions that increase someone's risk for cancer or heart disease.

Carry Forward Entered Choices - Entered Text from "Please describe up to five potential risks/harms of workplace wellness genetic testing. "

X→

Please click and drag to rank **up to five** potential **risks/harms** of workplace wellness genetic testing.

______

______

______

______

______

Please describe any other potential **risks/harms** of workplace wellness genetic testing.

____

____

____

End of Block: Risks/Harms

Start of Block: Design Features

In order to maximize the benefits you mentioned and minimize the risks you mentioned, what are the most important features to include when designing a workplace genetic testing program? Please describe **up to five** important **design features** to include for workplace wellness genetic testing.

- ○ ______________________________
- ○ ______________________________
- ○ ______________________________
- ○ ______________________________
- ○ ______________________________

Carry Forward Entered Choices - Entered Text from "In order to maximize the benefits you mentioned and minimize the risks you mentioned, what are the most important features to include when designing a workplace genetic testing program? Please describe up to five important design features to include for workplace wellness genetic testing."

X→

Please click and drag to rank **up to five** important **design features** to include for workplace wellness genetic testing.

______
______
______
______
______

Please write any other important **design features** to include for a workplace genetic testing program.

____
____
____
____
____

Workplace genetic testing: Voluntary genetic testing offered to employees as part of a workplace wellness program. This testing is not initiated by a provider and is not typically discussed in a visit with their clinician prior to testing. For example, a company may offer their employees the option to pursue genetic testing for inherited conditions that increase someone's risk for cancer or heart disease.

End of Block: Design Features

Start of Block: Agree/Disagree and Why/Why Not?

Please indicate whether you agree or disagree with the following statement

	Strongly agree	Somewhat Agree	Neither agree nor disagree	Somewhat disagree	Strongly disagree
Employers **should not be allowed** to offer voluntary workplace genetic testing.	○	○	○	○	○

Please indicate whether you agree or disagree with the following statement

	Strongly agree	Somewhat Agree	Neither agree nor disagree	Somewhat disagree	Strongly disagree
Employers **should offer** voluntary workplace genetic testing.	○	○	○	○	○

Please expand upon your above responses.

____

____

Workplace genetic testing: Voluntary genetic testing offered to employees as part of a workplace wellness program. This testing is not initiated by a provider and is not typically discussed in a visit with their clinician prior to testing. For example, a company may offer their employees the option to pursue genetic testing for inherited conditions that increase someone's risk for cancer or heart disease.

POST-WORKSHOP EVALUATION

Thank you for participating in the workshop! Please indicate your level of agreement with the following statements. Then, click the arrow below to continue.

Overall, I was satisfied with the interactive workshop.

- Strongly disagree
- Somewhat disagree
- Neither agree nor disagree
- Somewhat agree
- Strongly agree

My viewpoint was taken seriously during the workshop.

- Strongly disagree
- Somewhat disagree
- Neither agree nor disagree
- Somewhat agree
- Strongly agree

My opinions were respected by my small group.

- Strongly disagree
- Somewhat disagree
- Neither agree nor disagree
- Somewhat agree
- Strongly agree

My perspectives and participation made an impact on the interactive workshop.

- Strongly disagree
- Somewhat disagree
- Neither agree nor disagree
- Somewhat agree
- Strongly agree

There were diverse stakeholder perspectives at the workshop.

- Strongly disagree
- Somewhat disagree
- Neither agree nor disagree
- Somewhat agree
- Strongly agree

My time participating in this workshop was worthwhile.

- Strongly disagree
- Somewhat disagree
- Neither agree nor disagree
- Somewhat agree
- Strongly agree

I felt comfortable participating in the interactive workshop.

- Strongly disagree
- Somewhat disagree
- Neither agree nor disagree
- Somewhat agree
- Strongly agree

The presenters were unbiased on the topic.

- Strongly disagree
- Somewhat disagree
- Neither agree nor disagree
- Somewhat agree
- Strongly agree

I would abide by the group's final position, even if it differs from my personal opinion.

- Strongly disagree
- Somewhat disagree
- Neither agree nor disagree
- Somewhat agree
- Strongly agree

I felt that my group had a good discussion, even if I personally held a different viewpoint than my other small group members.

- Strongly disagree
- Somewhat disagree
- Neither agree nor disagree
- Somewhat agree
- Strongly agree

Differences in power between the stakeholders **negatively affected** the conversation in my small group.

- Strongly disagree
- Somewhat disagree
- Neither agree nor disagree
- Somewhat agree
- Strongly agree

Do you have any comments you wish to share about the workshop or this study, in general?

____

SURVEY 2

Of note, questions asked that did not pertain to the findings of this paper have been removed from this copy of Survey 2.

Start of Block: Introduction

Introduction
Thank you for your participation in our assessment of **workplace genetic testing**. To date, you have completed the first survey and participated in a deliberative workshop to identify potential **benefits and harms** of workplace genetic testing programs, as well as **design features** that could possibly maximize potential benefits while minimizing potential harms. For a refresher on workplace genetic testing, please watch the 4-minute video on the next page.

Please select "next page" to continue.

Page Break

https://www.youtube.com/watch?v=kyTCELnP3Bs

Please select "next page" to continue.

End of Block: Introduction

Start of Block: Section I: Revisiting the Initial Questions

Section I: Revisiting the Initial Survey Questions
In the first survey round, we asked participants to rate their agreement with the following statements, and the group provided us with the following responses:

Please select "next page" to continue.

Page Break

X→ X→

Using the scales provided, please **share your current opinion** by rating your agreement with these statements:

	Strongly disagree	Somewhat disagree	Neither agree nor disagree	Somewhat agree	Strongly agree
Employers **should offer** workplace genetic testing.	○	○	○	○	○
Employers **should not be allowed** to offer workplace genetic testing.	○	○	○	○	○
Employers **should have the option** to offer workplace genetic testing.	○	○	○	○	○

Please select "next page" to continue.

Page Break

How have your thoughts about these questions changed since the first survey, if at all? Why or why not?

____

____

____

____

____

Please select "next page" to continue.

Page Break

X→

End of Block: Section I: Revisiting the Initial Questions

Start of Block: Section II: Key Characteristics Intro

Section II: 12 Key Characteristics Based on the initial survey and the deliberative workshop, the participants of this study identified **12 key characteristics** that could potentially maximize benefits and minimize harms of workplace genetic testing.

Using the scales provided, **please rate your agreement** with the statements on the next pages about the importance, likelihood of being achieved, and necessity of the **12 key characteristics** for maximizing potential benefits and minimizing potential harms of workplace genetic testing programs.

As an individual participant in this study, you may or may not agree on the key characteristics identified by the study participants as a whole. In this second survey, we hope to gain a better understanding of where study participants **share common ground.**

Please select "next page" to continue.

End of Block: Section II: Key Characteristics Intro

Start of Block: Section II: Employee Control

Employee control: Measures to ensure employees are in charge of the management and use of data from workplace genetic testing.

	Strongly disagree	Somewhat disagree	Neither agree nor disagree	Somewhat agree	Strongly agree
Employee control is **important.**	○	○	○	○	○
Employee control is **likely to be achieved.**	○	○	○	○	○

Employee control is **necessary** in order for workplace genetic testing to be offered.

○ No, I disagree.

○ Yes, I agree.

Please select "next page" to continue.

End of Block: Section II: Employee Control

Start of Block: Section II: Transparency

Transparency: Measures to ensure there is clear communication about how the workplace genetic testing program works - the process and data protections.

	Strongly disagree	Somewhat disagree	Neither agree nor disagree	Somewhat agree	Strongly agree
Transparency is **important.**	○	○	○	○	○
Transparency is **likely to be achieved.**	○	○	○	○	○

Transparency is **necessary** in order for workplace genetic testing to be offered.

- No, I disagree.
- Yes, I agree.

Please select "next page" to continue.

End of Block: Section II: Transparency

Start of Block: Section II: Anti-Discrimination

Anti-discrimination: Measures to prevent unfair treatment based on data from workplace genetic testing.

	Strongly disagree	Somewhat disagree	Neither agree nor disagree	Somewhat agree	Strongly agree
Anti-discrimination is **important.**	○	○	○	○	○
Anti-discrimination is **likely to be achieved.**	○	○	○	○	○

Anti-discrimination is **necessary** in order for workplace genetic testing to be offered.

- No, I disagree.
- Yes, I agree.

Please select "next page" to continue.

End of Block: Section II: Anti-Discrimination

Start of Block: Section II: Privacy/Security

Privacy/security: Measures to ensure the confidentiality and security of workplace genetic test results.

	Strongly disagree	Somewhat disagree	Neither agree nor disagree	Somewhat agree	Strongly agree
Privacy/security is **important.**	○	○	○	○	○
Privacy/security is **likely to be achieved.**	○	○	○	○	○

Privacy/security is **necessary** in order for workplace genetic testing to be offered.

○ No, I disagree.

○ Yes, I agree.

Please select "next page" to continue.

End of Block: Section II: Privacy/Security

Start of Block: Section II: Voluntariness

Voluntariness: Measures to prevent coercion or pressure to participate in workplace genetic testing.

	Strongly disagree	Somewhat disagree	Neither agree nor disagree	Somewhat agree	Strongly agree
Voluntariness is **important.**	○	○	○	○	○
Voluntariness is **likely to be achieved.**	○	○	○	○	○

Voluntariness is **necessary** in order for workplace genetic testing to be offered.

○ No, I disagree.

○ Yes, I agree.

Please select "next page" to continue.

End of Block: Section II: Voluntariness

Start of Block: Section II: Understanding/Education

Understanding/education: Measures to ensure employees have a good understanding of workplace genetic testing before and after the test (for example: education, genetic counseling).

	Strongly disagree	Somewhat disagree	Neither agree nor disagree	Somewhat agree	Strongly agree
Understanding/education is **important.**	○	○	○	○	○
Understanding/education is **likely to be achieved.**	○	○	○	○	○

Understanding/education is **necessary** in order for workplace genetic testing to be offered.

- No, I disagree.
- Yes, I agree.

Please select "next page" to continue.

End of Block: Section II: Understanding/Education

Start of Block: Section II: Equity

Equity: Measures to ensure there is equitable access to workplace genetic testing, related benefits, and follow-up.

	Strongly disagree	Somewhat disagree	Neither agree nor disagree	Somewhat agree	Strongly agree
Equity is **important.**	○	○	○	○	○
Equity is **likely to be achieved.**	○	○	○	○	○

Equity is **necessary** in order for workplace genetic testing to be offered.

- No, I disagree.
- Yes, I agree.

Please select "next page" to continue.

End of Block: Section II: Equity

Start of Block: Section II: EBT

X→

Evidence-based testing: Measures to ensure testing is performed by a reputable laboratory and that what is analyzed on the test is supported by evidence and produces accurate results.

	Strongly disagree	Somewhat disagree	Neither agree nor disagree	Somewhat agree	Strongly agree
Evidence-based testing is **important.**	○	○	○	○	○
Evidence-based testing is **likely to be achieved.**	○	○	○	○	○

X→

Evidence-based testing is **necessary** in order for workplace genetic testing to be offered.

○ No, I disagree.

○ Yes, I agree.

Please select "next page" to continue.

End of Block: Section II: EBT

Start of Block: Section II: Health care integration

X→

Health care integration: Measures to ensure that workplace genetic test results can be integrated into the healthcare system and/or the electronic medical record.

	Strongly disagree	Somewhat disagree	Neither agree nor disagree	Somewhat agree	Strongly agree
Health care integration is **important.**	○	○	○	○	○
Health care integration is **likely to be achieved.**	○	○	○	○	○

Health care integration is **necessary** in order for workplace genetic testing to be offered.

○ No, I disagree.

○ Yes, I agree.

Please select "next page" to continue.

End of Block: Section II: Health care integration

Start of Block: Section II: User-friendliness

User-friendliness: Measures to ensure the workplace genetic testing program has a simple and easy-to-use design.

	Strongly disagree	Somewhat disagree	Neither agree nor disagree	Somewhat agree	Strongly agree
User-friendliness is **important.**	○	○	○	○	○
User-friendliness is **likely to be achieved.**	○	○	○	○	○

User-friendliness is **necessary** in order for workplace genetic testing to be offered.

- No, I disagree.
- Yes, I agree.

Please select "next page" to continue.

End of Block: Section II: User-friendliness

Start of Block: Section II: Utility for employers

Utility for employers: Measures to ensure **employers** can reap benefits through offering workplace genetic testing to their employees

	Strongly disagree	Somewhat disagree	Neither agree nor disagree	Somewhat agree	Strongly agree
Utility for employers is **important.**	○	○	○	○	○
Utility for employers is **likely to be achieved.**	○	○	○	○	○

Utility for employers is **necessary** in order for workplace genetic testing to be offered.

- No, I disagree.
- Yes, I agree.

Please select "next page" to continue.

End of Block: Section II: Utility for employers

Start of Block: Section II: Utility for laboratories

X→

Utility for laboratories: Measures to ensure **laboratories** can reap the benefits of performing and/or offering services related to workplace genetic testing.

	Strongly disagree	Somewhat disagree	Neither agree nor disagree	Somewhat agree	Strongly agree
Utility for laboratories is **important.**	○	○	○	○	○
Utility for laboratories is **likely to be achieved.**	○	○	○	○	○

X→

Utility for laboratories is **necessary** in order for workplace genetic testing to be offered.

○ No, I disagree.

○ Yes, I agree.

Please select "next page" to continue.

End of Block: Section II: Utility for laboratories

Start of Block: Section III: Final Questions

X→ X→

Assuming the key characteristics you described as NECESSARY were met, please now rate your agreement with the following statements:

	Strongly disagree	Somewhat disagree	Neither agree nor disagree	Somewhat agree	Strongly agree
Employers **should offer** workplace genetic testing.	○	○	○	○	○
Employers **should not be allowed** to offer workplace genetic testing.	○	○	○	○	○
Employers **should have the option** to offer workplace genetic testing.	○	○	○	○	○

Please select "next page" to continue.

Start of Block: Thank you!

Thank you for participating in our study. You will receive your Amazon e-gift cards in the amount of $25 for completing this survey and $100 for completing all three steps of our study within the next month.

End of Block: Thank you!

MODIFIED DELPHI PROCESS METHODS

Participants and recruitment

List of potential stakeholders

The research team (E.C., W.G.F., A.E.R.P., K.R., K.S., A.V.) developed a list of potential stakeholders for participation in the study from important stakeholder groups through an iterative process: employees (full-time workers and/or labor organization representatives), employers (managers of companies and/or business organization representatives), healthcare professionals (including healthcare organization representatives and clinicians), genetic testing

industry or insurance professionals (health, life, or disability insurance), and policy experts or ELSI professionals (bioethics, legal, or privacy experts and/or policy makers). The research team compiled a list of potential stakeholders organized by stakeholder category, including individuals and key organizations with publicly available contact information. The broader R01 research team and advisory council representing the stakeholder categories provided additional potential stakeholders. Potential stakeholders for participation in the study were prioritized based on types of perspectives they might represent as well as demographic variables such as years of experience, education, race/ethnicity, gender to try to maximize perspectives represented within stakeholder groups.

Screening potential stakeholders for study participation

Personalized invitations were sent up to three times per potential stakeholder, per online platform (e.g., email, LinkedIn). The screener (programmed in Qualtrics) identified potential stakeholders' self-reported stakeholder category from among the curated list (e.g., employees, employers, ELSI professionals, genetic testing industry representatives, and healthcare professionals). The screener also collected self-reported gender, race and ethnicity, and US region through multiple-response (select-all-that-apply) questions and perspectives on the selected stakeholder category through free-text responses. Given the broad spectrum of potential stakeholders in the "employee" stakeholder category, an additional approach was utilized to screen employees. The University of Michigan Health Research website (umhealthresearch.org) was used to advertise the study, and included a link to the study screener. This secure, password-protected database has a pool of over 90,000 individuals who have consented to be contacted for research opportunities.

The study invitation and the screener facilitated snowball recruitment of additional experts by forwarding the invitation within their professional network and soliciting contact information for potential stakeholders respectively. Respondents to the screener needed to be fluent in English, aged 18 years or older, have access to a computer or tablet that allowed video

conferencing, have internet access, and belong to one or more of the stakeholder categories to be eligible to participate in this study.

Selecting study invitees and final study participants

The research team that included a primary care provider, legal scholar, qualitative researcher, and genetic counselors selected the final list of potential stakeholders to be invited to participate in the study. The study consent form which detailed the entire modified Delphi process including the immediate next step of administering Survey 1 was sent by email to the selected study invitees. Those who completed the consent form followed by Survey 1 were considered to be enrolled study participants.

Modified Delphi Procedure and Materials

Survey 1 (June 2023)

Each participant completed a 20-minute online initial mixed-methods survey about their individual perspectives regarding employers offering genetic testing. Survey 1 began with an informational video created by the genetic counselor on the research team (E.C.) to introduce participants to the concept of wGT. We ascertained primary stakeholder role, years of experience, highest level of education, and age. Participants were then asked to list and rank, through free-text responses, up to five (a) potential benefits of wGT, (b) potential harms of wGT, and (c) potential design features important to consider for wGT. Survey 1 also included two Likert-type scale questions to assess participants' level of agreement on a 5-point scale (*1=Strongly agree, 5=Strongly disagree*) with the following statements: "*Employers should offer workplace genetic testing*" and "*Employers should NOT be allowed to offer workplace genetic testing*."

Deliberative Workshop (September 2023)

The 5-hour online Deliberative Workshop included three plenary sessions and two break-out (small group) discussions.

<u>Plenary session 1: Expert presentations</u>.

In the first plenary session, W.G.F. and A.E.R.P. (experts) provided educational presentations on workplace wellness programs, wGT, and relevant laws such as the Genetic Information Nondiscrimination Act and the Americans with Disabilities Act. They also shared the aggregate, de-identified results of Survey 1 with participants, including the top ranked benefits, harms, and design features of wGT. Each presentation was followed by a brief discussion/question and answer period and provided context for the subsequent small group session. The experts were also available to answer questions during the small group discussions if requested by the small group.

Small group discussions. Participants were assigned to one of five small groups led by a trained facilitator. Small groups were broadly segregated by stakeholder groups (employee, employer, ELSI scholar, genetic testing industry representative, healthcare professional). Prior to the workshop, facilitators attended a 2-hour training session with the research team that provided an overview of the research topic, the modified Delphi process, the Deliberative Workshop agenda, and a review of the small group discussions that they would be facilitating. The first small group discussion focused on the benefits and harms of wGT. Participants were shown Survey 1 qualitative data on the proposed or ranked benefits and harms of wGT to deliberate in small groups. Similarly, the second small group discussion focused on design features for wGT.

Plenary Sessions 2 and 3. In the second and third plenary sessions, each trained facilitator provided a summary of the content of their assigned small group's discussion. During the second plenary session, participants were given an opportunity to ask additional questions and provide responses to the report-backs. During the third plenary session, participants were shown data in aggregate from Survey 1 regarding the Likert-type scale questions. They were then asked to provide further commentary on their views and how their perspectives may have changed since the time of Survey 1. A.E.R.P. and W.G.F. moderated the discussion for plenary sessions 2 and 3.

Post-workshop evaluation. A short post-workshop evaluation was administered to determine if group dynamics could have impacted the results of the workshop.

Survey 2 (April 2024)

Survey 2 included the introductory YouTube video (shown in Survey 1). Participants were then asked to view the aggregate data from Survey 1 and re-rate their agreement with statements about employers offering/being allowed to offer wGT based on their current opinion. Participants were then asked to rate their agreement with five statements on secondary use of wGT results. Next, participants were asked to rate their agreement on statements on wGT about the importance and likelihood of being achieved. All agreement ratings were based on a 5-point scale that ranged from 1=*Strongly agree* to 5=*Strongly disagree*. For assessing participants' agreement on necessity (in order for wGT to be offered) for each of twelve "key characteristics" that could potentially maximize the benefits and minimize the harms of wGT, we deployed a binary response option (*1=Yes, I agree, 2=No, I disagree*). The 12 key characteristics for assessment were informed by findings from the deliberative workshop. Finally, participants were asked to again answer the questions about employers offering/being allowed to offer wGT, assuming the key characteristics they described as "necessary" in order for wGT to be offered were met.

Data Analysis

Survey Analysis

For Survey 1, each research team member reviewed up to five initial survey responses and made note of themes for the purpose of codebook development. Responses pertaining to benefits, risks and design features of wGT were included in the initial review and themes relevant to the key research questions were included in the codebook. The codebook was then comprehensively reviewed by the entire research team multiple times to ensure appropriate focus, accuracy. Benefits, risks, and design features of wGT were thematically coded in real-time by a qualitative researcher (K.R.) and a genetic counselor (E.C.) during an online team

meeting using MAXQDA software (VERBI Software, 2021). A lawyer with expertise in health law and ELSI (A.E.R.P.) then provided a final review of responses that were unclear or fell into an uncaptured theme. Furthermore, a single coder (E.C.) applied rank codes (1-5) based on survey responses, and overlap among the thematic codes and the rank codes was reviewed by the research team to determine which themes were most frequently ranked 1-5 for benefits, risks and design features. Data generated from this analysis was shown to participants during the deliberative workshop.

The rest of the quantitative survey data from Survey 1 and Survey 2 of the modified Delphi process were summarized using descriptive statistics. A biostatistician (S.P.) applied Chi-square tests to analyze differences between stakeholder groups with respect to (a) agreement with the necessity of each of the 12 key characteristics, and (b) distribution of agreement items about employers offering/being allowed to offer wGT from the first portion of Survey 2.

We undertook several types of analyses to track participant responses to the questions about whether employers should offer/be allowed to offer wGT. Descriptively, we created five categories of responses based on the Likert-type scale scores for the two normative questions: 1) supportive, 2) permissive, 3) neutral/conflicted, 4) libertarian, and 5) opposed. These categories were finalized by comparing both quantitative Likert-type scale scores to qualitative open-ended responses. We then assessed the extent to which participants changed their opinions across Survey 1, the beginning of Survey 2, and the end of Survey 2 (when the design features for wGT were assumed). Furthermore, Chi-square analysis was used to determine if there were significant differences between participants' likelihood of moving towards agreement or disagreement with questions about employers offering/being allowed to offer wGT from (a) Survey 1 (pre-workshop) to the beginning of Survey 2 (post-workshop), (b) Survey 1 (pre-workshop) to the end of Survey 2 (post-workshop, assuming key characteristics met), and c) the

beginning of Survey 2 (post-workshop) to the end of Survey 2 (post-workshop, assuming key characteristics met).

Deliberative Workshop Analysis

Audio recordings of the small group discussions and plenary sessions were transcribed verbatim and de-identified by LandMark Associates, a HIPAA-compliant transcribing service. Each research team member (A.E.R.P., W.G.F., K.R., K.S., E.C.) reviewed 2-4 transcripts each, and created a memo for each transcript. Memos were reviewed with attention to recommendations for wGT design features and key characteristics for implementation of wGT. From the memos, twelve “key characteristics” for maximizing the benefits and minimizing the potential harms of wGT were identified. Representative quotes were collected by K.R. for each key characteristic.

SUPPLEMENTAL TABLES AND FIGURES

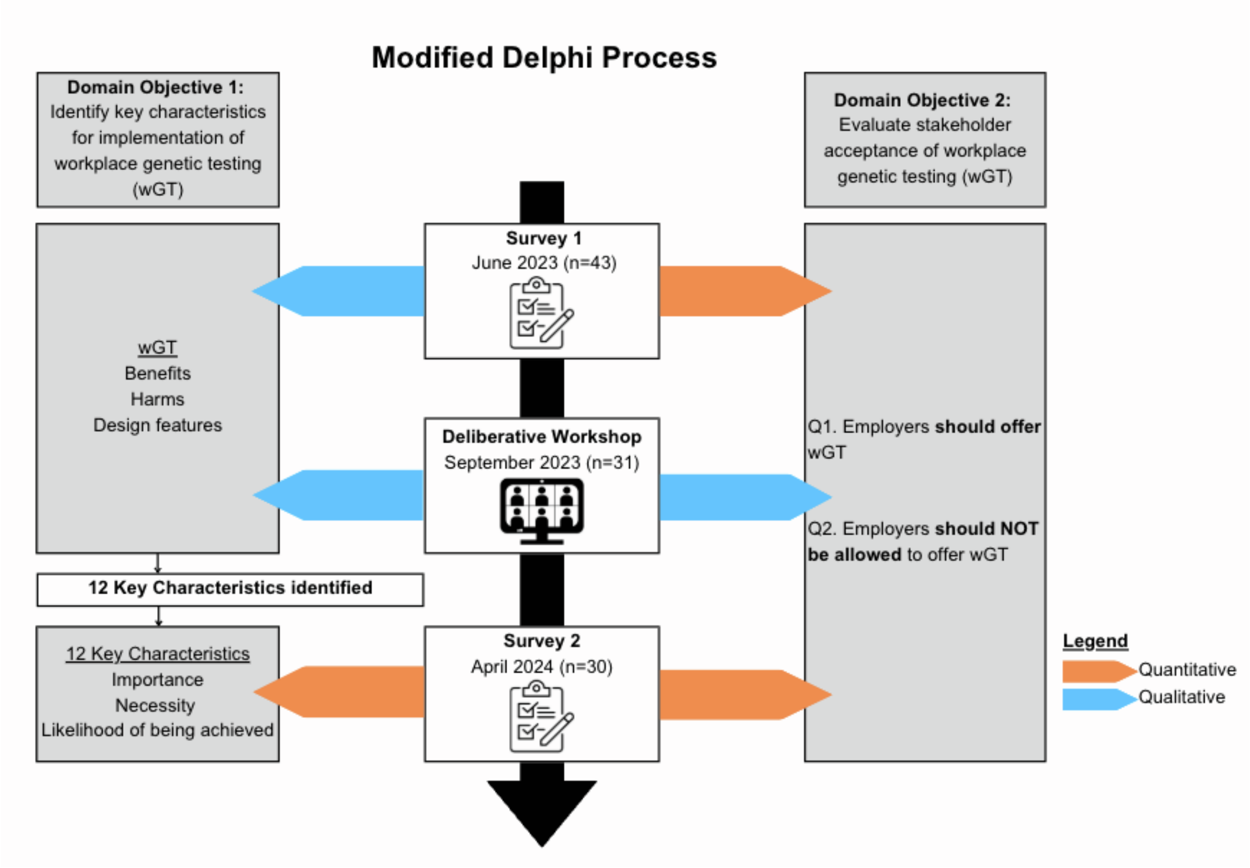

Supplemental Figure 1. Overview of the modified Delphi process. Important stakeholder perspectives on (1) key characteristics for implementation of workplace genetic testing (wGT) and (2) acceptance of wGT were evaluated through a three-round, mixed-methods Delphi process including Survey 1, a Deliberative Workshop, and Survey 2 (See Methods and additional details about methods described in Supplemental Materials).

Supplemental Table 1. Summary of research participants responding through the modified Delphi process.

Stakeholder Category	N (%)<br>Survey 1<br>(N=43)	N (%)<br>Deliberative Workshop<br>(N=31)	N (%)<br>Survey 2<br>(N=30)
Employee	8 (19%)	6 (19%)	6 (20%)
Employer	8 (19%)	4 (13%)	4 (13%)
ELSI professional	10 (23%)	9 (29%)	8 (27%)
Genetic testing company/ industry representative	8 (19%)	8 (26%)	8 (27%)
Healthcare professional	9 (21%)	4 (13%)	4 (13%)

Supplemental Table 2. Benefits and risks for workplace genetic testing (wGT) emerging from participants' free-text responses from Survey 1 (codebook for Survey 1) (n=43).

Benefits	Definition
Top benefits	
Access	Any mention of improved access to genetic services or testing, including through the reduction of barriers, increased convenience; Genetic testing leading to access to support or resources; Making it easier to receive genetic testing; convenient access
Health impacts	Any mention of changing employee healthcare behaviors, informing employees that they need to engage in healthcare behaviors (such as screening, testing, medication change, etc.), or engagement with healthcare providers; proactive in healthcare system OR any mention of positive health impact, health improvement or improved clinical outcome for employee; personalized or targeted therapy; Preventing negative health outcomes; Early detection and intervention
Knowledge	Any mention of employee having a greater awareness or knowledge of genetics, genetic risk, "knowledge is power" sentiments; Increasing knowledge of genetics in general; Awareness or curiosity
Some mentioned	
Family health impacts	Any mention of positive health impact for the participant's family, including cascade testing or screening, prevention, planning, etc.; Family receiving information about health and risks; reproductive implications of genetic testing
Positive emotions	Any mention of positive employee reactions, such as peace of mind, reduced anxiety, increased satisfaction, feeling motivated, relief, etc.
(Reduced) cost	Any mention of being free or low cost to employee; discounted cost; reduced insurance premiums, and incentives that reduce cost
Few discussed	
De-stigmatization	Any mention of normalizing or destigmatizing genetic testing or participating in genetic testing
Financial planning	Any mention of being proactive/planning for the future (i.e., finances, disability, insurance) and explicit reference to expenses related to health
Lifestyle/behavior impacts	Any mention of changing employee lifestyle behaviors, or informing employee that they need to engage in lifestyle behaviors (such as diet, exercise, reducing stress, etc.); proactive in lifestyle changed
Employee recruitment and retention	Any mention of increasing employee satisfaction or loyalty; demonstrating that the employer cares about employees; retaining employees OR any mention of wGT being a differentiator or an enticing option for potential employees, improving recruitment or recruitment advantage
Reducing employer costs	Any mention of reducing costs; reducing insurance costs to employer; reducing absenteeism/sick days; healthier employees leading reduced costs
Business planning	Any mention of a benefit to the employer for business planning purposes, including benefits planning, or preparing for future business or benefits needs
**Risks**	**Definition**
Top risks	
Discrimination	Any mention of employees experiencing insurance discrimination (health, disability, life insurance), increased insurance cost or denial of coverage OR any mention of employees experiencing employment discrimination; Not receiving promotion; Not being hired; being treated unfairly by employers

Lack of access to follow-up	Any mention of employees either lacking access to follow-up, being unable to afford follow-up, not knowing what to do for follow-up healthcare or genetic counseling, or receiving incorrect or costly follow-up care; Lack of guidance regarding follow-up care; Lack of or inadequate post-test genetic counseling
Lack of understanding	Any mention of employees receiving inadequate education or lack of understanding of genetics, genetic testing, or genetic results, or misunderstandings; False sense of security; Genetic determinism; Results are too complicated to understand; inadequate or lack of pretest education/counseling
Negative emotions	Any mention of negative employee reactions, such as anxiety, depression, hopelessness, feeling stigmatized, traumatized by results, uncertainty, etc.
Privacy-related risks	Any mention of employee concerns about privacy, confidentiality, and invasions of privacy. Who has access to the data by design?
Some mentioned	
Coercion	Any mention of a employees feeling coerced or pressured into genetic testing by the employer
Health mismanagement	Any reference to unnecessary or harmful medical screenings, surveillance, surgeries, etc. based on results; Inappropriate lifestyle or health decision-making by patient or provider
Inaccurate testing	Any mention of employees receiving or misleading or inaccurate results; False positive or false negatives
Security-related risks	Any mention of hacking, leaked data, encryption, protocols for ensuring security of data; stopping unintended access
Few discussed	
Inconclusive results/VUS	Any mention of VUS (variants of uncertain significance) or inconclusive genetic test results
Lack of test actionability	Any discussion of lack of evidence for testing or no ability to act on results
Risks to employees' family members	Any discussion of family in the risks section, including risk to family
Risks to the employer	Any mention of negative impact to the employers, including sunk investment in the program, cost, or low participation, liability, lower employee satisfaction or trust
Secondary use of data	Any mention of secondary use by a third party or selling of employee data; Lack of control by employee of what happens to their data; misuse of data by employers if discrimination was not specifically mentioned
Stigma	Any mention of stigma, loss of reputation, or being looked down upon for genetic testing, participating in genetic testing, or genetic testing results

Supplemental Table 3. Results of post-workshop evaluation (n=29).

Statement	N (%)				
	Strongly Agree	Somewhat Agree	Neither Agree nor Disagree	Somewhat Disagree	Strongly Disagree
Overall, I was satisfied with the interactive workshop.	16 (55%)	10 (34%)	--	2 (7%)	1 (3%)
My viewpoint was taken seriously during the workshop.	25 (86%)	2 (7%)	--	1 (3%)	1 (3%)
My opinions were respected by my small group.	26 (90%)	2 (7%)	--	1 (3%)	--
My perspectives and participation made an impact on the interactive workshop.	15 (52%)	10 (34%)	2 (7%)	1 (3%)	1 (3%)
There were diverse stakeholder perspectives at the workshop.	17 (59%)	8 (28%)	2 (7%)	1 (3%)	1 (3%)
My time participating in this workshop was worthwhile.	15 (52%)	11 (38%)	3 (10%)	--	--
I felt comfortable participating in the interactive workshop.	23 (79%)	4 (14%)	2 (7%)	--	--
The presenters were unbiased on the topic.	26 (90%)	1 (3%)	1 (3%)	1 (3%)	--
I would abide by the group's final decision, even if it differs from my personal opinion.	6 (21%)	5 (17%)	10 (34%)	5 (17%)	3 (10%)
I felt that my group had a good discussion, even if I personally held a different viewpoint than my other small group members.	25 (86%)	3 (10%)	1 (3%)	--	--
Differences in power between the stakeholders negatively affected the conversation in my small group.	--	--	4 (14%)	4 (14%)	21 (72%)

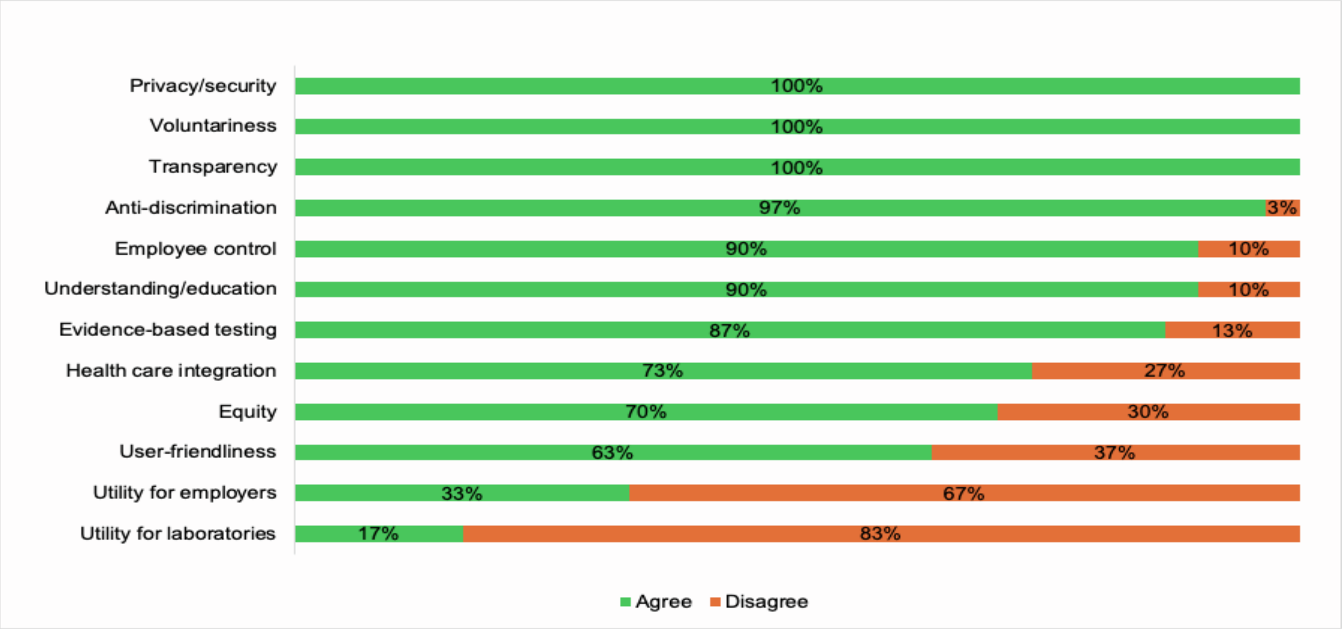

Supplemental Figure 2. Participant perspectives on the necessity of key characteristics in order for wGT to be offered (n=30).

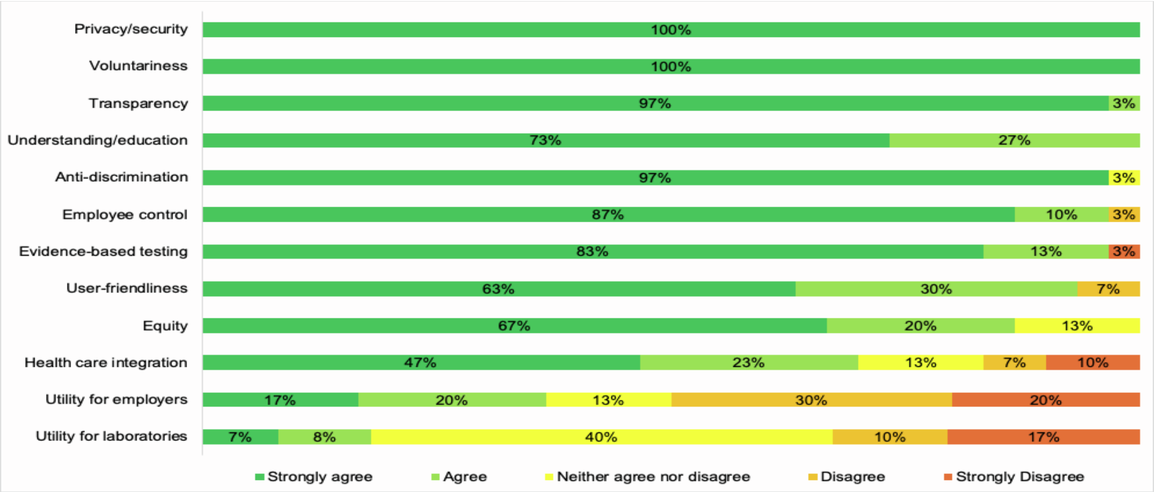

Supplemental Figure 3. Participant perspectives on the <u>importance</u> of key characteristics for wGT (n=30).

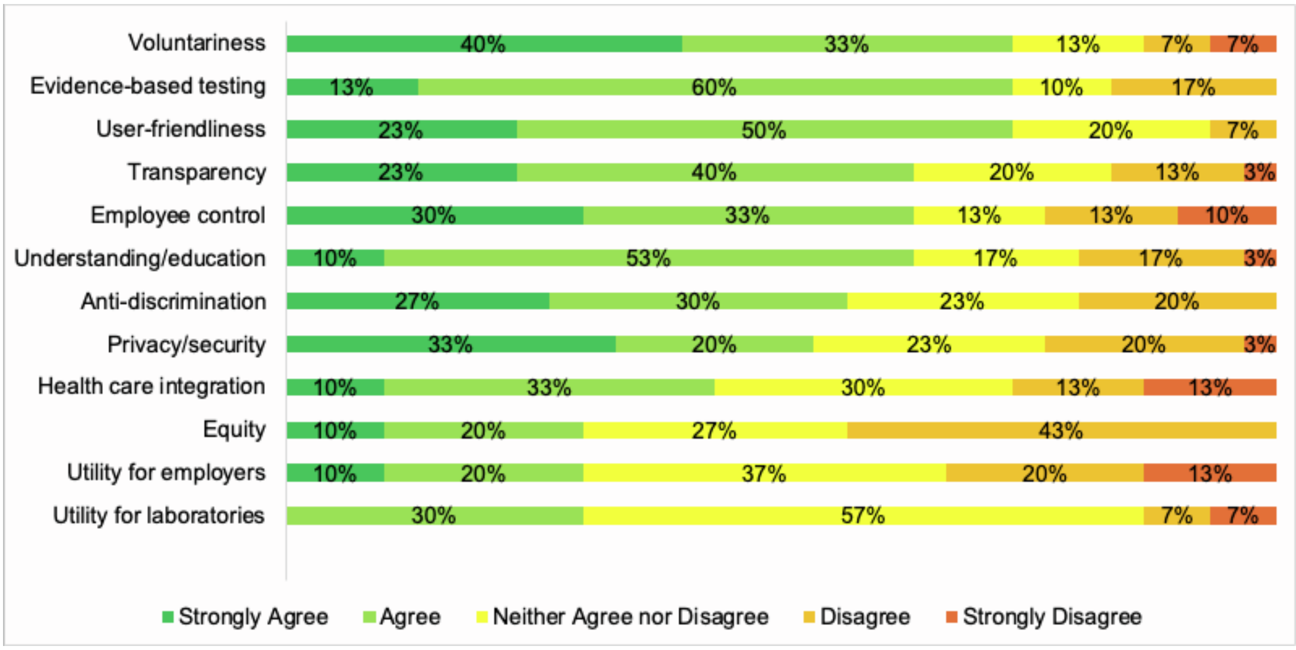

Supplemental Figure 4. Participant perspectives on the likelihood of key characteristics for wGT being achieved (n=30).
